# Thermodynamic Implementations of Quantum Processes

**DOI:** 10.1007/s00220-021-04107-w

**Published:** 2021-05-28

**Authors:** Philippe Faist, Mario Berta, Fernando G. S. L. Brandao

**Affiliations:** 1 Institute for Quantum Information and Matter, Caltech, Pasadena, CA 91125 USA; 2 Institute for Theoretical Physics, ETH Zurich, 8093 Zurich, Switzerland; 3 Dahlem Center for Complex Quantum Systems, Freie Universität Berlin, 14195 Berlin, Germany; 4 Department of Computing, Imperial College London, London, SW7 2AZ UK; 5 AWS Center for Quantum Computing, Pasadena, CA 91125 USA

## Abstract

Recent understanding of the thermodynamics of small-scale systems have enabled the characterization of the thermodynamic requirements of implementing quantum processes for fixed input states. Here, we extend these results to construct optimal universal implementations of a given process, that is, implementations that are accurate for any possible input state even after many independent and identically distributed (i.i.d.) repetitions of the process. We find that the optimal work cost rate of such an implementation is given by the thermodynamic capacity of the process, which is a single-letter and additive quantity defined as the maximal difference in relative entropy to the thermal state between the input and the output of the channel. Beyond being a thermodynamic analogue of the reverse Shannon theorem for quantum channels, our results introduce a new notion of quantum typicality and present a thermodynamic application of convex-split methods.

## Introduction

In the information-theoretic approach to thermodynamics, a careful analysis of the resources required to perform thermodynamic tasks has allowed to consistently and systematically describe the thermodynamic behaviour of quantum systems at the nano-scale [1]. In particular, thermodynamics can be phrased as a resource theory [2–4]. In a resource theory, one specifies which operations can be carried out at no cost—the *free operations*—and then one studies how much of external resources (e.g., thermodynamic work) one needs to provide to carry out operations that are not free. Two established resource theories for quantum thermodynamics are *thermal operations* [2, 3] and *Gibbs-preserving maps* [5, 6]. In the former, the free operations consist of energy-conserving interactions of the system with a heat bath, while in the latter, the free operations are any quantum operation that preserves the thermal state. It is reasonable to assume that thermal operations can be realized in an idealized setting, making them a good choice of framework for constructing explicit protocols, whereas Gibbs-preserving maps encompass a broader class of operations, allowing us to derive stronger fundamental limits.

The resource theory approach to thermodynamics has revealed close connections with measures of information known from quantum information theory [7, 8]. Namely, single-shot thermodynamic and information-theoretic tasks are both quantified by relevant entropy measures [9–11]. Consequently, tools from quantum Shannon theory can be used to characterize tasks in thermodynamics, for instance to derive second-order asymptotics of the work cost of state transformations [12]. Recently, focus was shifted to understand the resource costs of quantum processes, rather than state transformations [13–16]. The information measure associated with quantum processes is the quantum capacity, along with its many variants [17]. A natural question arises: What is the thermodynamic analogue of the quantum capacity?

Here, we ask how much work is required to implement a given quantum process, with the requirement that the implementation is accurate for any possible input state. In the single-instance regime, we find that the answer is a variation of the results obtained in Ref. [16]. However, in the regime where we consider many independent and identically distributed (i.i.d.) copies of the process, important differences arise due to typicality. We find that the optimal work cost of such an implementation in the i.i.d. regime is given by the thermodynamic capacity, defined as the maximal difference between the input and output free energy of the process over all possible input states. The fact that no implementation can perform better than the thermodynamic capacity follows fairly straightforwardly from the results of Ref. [16]. The technically challenging part of the present paper is to show that there exist protocols that achieve this limit.

We provide three different constructions of such protocols, each valid in different settings. In the first construction, we make the simplifying assumption that Hamiltonian of the system is trivial as in Ref. [13]. We then show that simple properties of one-shot entropy measures, coupled with the post-selection technique [18], provide an existence proof of the required implementation. The implementation is given in terms of thermal operations. In our second construction, we develop novel quantum typicality tools which we use along with the post-selection technique to explicitly construct an implementation in terms of Gibbs-preserving maps for any i.i.d. process and for any system Hamiltonian. In our third construction, we assume that the i.i.d. process is time-covariant, i.e., commutes with the time evolution. We then use recent results on the convex-split lemma and position-based decoding [19] to construct an implementation of a time-covariant i.i.d. process with thermal operations.

Our results imply that the thermodynamic resource theory of channels becomes reversible in the i.i.d. limit [20]. Namely, invoking the results in Ref. [21], we see that the work rate that is required to implement a given i.i.d. process is the same as what can be extracted if the i.i.d. process is provided to us as a black box. This provides a thermodynamic analogue of the reverse Shannon theorem from quantum information theory. This theorem states that the quantum mutual information of the channel uniquely characterizes the resources required to simulate the channel with noiseless channel uses and shared entanglement, as well as to distil a noiseless channel from many uses of the channel and shared entanglement [22, 23]. Indeed, our proof techniques are inspired by Refs. [22, 24–26].

The remainder of this paper is structured as follows. Section 2 gives the necessary preliminaries and fixes some notation. Section 3 introduces two resource theories for thermodynamics, thermal operations and Gibbs-preserving maps. In Sect. 4 we introduce the thermodynamic capacity and present some elementary properties. In Sect. 5, we provide our first construction for a trivial Hamiltonian. In Sect. 6 we provide our second construction, which is valid in the general setting and provides an implementation in terms of Gibbs-preserving maps. Section 7 provides our third construction, valid for time-covariant i.i.d. processes, and built with thermal operations. Our conclusions are presented in Sect. 8. Various more technical proof details are deferred to “Appendices A–F”.

## Preliminaries

### Quantum states, quantum processes, and distance measures

Each quantum system considered lives in a finite-dimensional Hilbert space. A quantum state is a positive semi-definite operator $\rho$ satisfying ${\text {tr}}[\rho ]=1$. A sub-normalized quantum state is a positive semi-definite operator $\rho$ satisfying ${\text {tr}}[\rho ]\leqslant 1$. To each system *S* is associated a standard basis, usually denoted by $\{ |{k}\rangle _S \}$. For any two systems $A,A'$, we denote by $A\simeq A'$ the fact that they are isometric. In that case, we consider a representation in which the isometry maps the standard basis onto the standard basis, i.e., ${\mathrm {id}}_{A\rightarrow A'}(|{k}\rangle \langle {k}|_A) = |{k}\rangle \langle {k}|_{A'}$ for all *k*, where ${\mathrm {id}}_{A\rightarrow A'}$ denotes the identity process. For any two systems $A\simeq A'$, we define the non-normalized maximally entangled reference ket $|{\varPhi }\rangle _{A:A'} = \sum _k\, |{k}\rangle _A\otimes |{k}\rangle _{A'}$. Matrix inequalities are with respect to the positive semi-definite cone: $A\leqslant B$ signifies that $B-A$ is positive semi-definite. A completely positive map ${\mathscr {E}}_{X\rightarrow X'}$ is a linear mapping that maps Hermitian operators on *X* to Hermitian operators on $X'$ and that satisfies ${\mathscr {E}}_{X\rightarrow X'}(\varPhi _{X:R_X}) \geqslant 0$, where $R_X\simeq X$. The adjoint ${\mathscr {E}}_{X\leftarrow X'}^\dagger$ of a completely positive map ${\mathscr {E}}_{X\rightarrow X'}$ is the unique completely positive map $X'\rightarrow X$ that satisfies ${\text {tr}}[{\mathscr {E}}(Y)\,Z] = {\text {tr}}[Y{\mathscr {E}}^\dagger (Z)]$ for all operators *Y*, *Z*. A completely positive map ${\mathscr {E}}_{X\rightarrow X'}$ is trace-preserving if ${\mathscr {E}}^\dagger (\mathbb {1}_{X'}) = \mathbb {1}_X$ and trace non-increasing if ${\mathscr {E}}^\dagger (\mathbb {1}_{X'}) \leqslant \mathbb {1}_X$.

Proximity of quantum states can be measured by the fidelity $F(\rho ,\sigma ) = \Vert {\sqrt{\rho }\sqrt{\sigma }}\Vert _1$, where the one-norm of an operator is defined as $\Vert {A}\Vert _1 = {\text {tr}}\big [\sqrt{A^\dagger A}\big ]$. The fidelity is extended to sub-normalized states $\rho ,\sigma$ as the generalized fidelity, ${\bar{F}}(\rho ,\sigma ) = \Vert {\sqrt{\rho }\sqrt{\sigma }}\Vert _1 +\sqrt{(1-{\text {tr}}[\rho ])(1-{\text {tr}}[\sigma ])}$, noting that $F(\cdot ,\cdot ) = {\bar{F}}(\cdot ,\cdot )$ whenever at least one of the states is normalized. An associated metric can be defined for any sub-normalized states as $P(\rho ,\sigma ) = \sqrt{1-{\bar{F}}^2(\rho ,\sigma )}$, called the purified distance [10, 11, 27], or root infidelity, and is closely related to the Bures distance and the quantum angle [28]. The proximity of two sub-normalized quantum states $\rho ,\sigma$ may also be measured in the trace distance $D(\rho ,\sigma ) = \frac{1}{2}\Vert {\rho -\sigma }\Vert _1$. We note that the one-norm of a Hermitian operator *A* can be expressed as

1 $$\begin{aligned} \Vert {A}\Vert _1 = \max _{\Vert {Z}\Vert _\infty \leqslant 1} {\text {tr}}[ZA]= \min _{\begin{array}{c} \varDelta _\pm \geqslant 0\\ A = \varDelta _+ - \varDelta _- \end{array}}{\text {tr}}[\varDelta _+] + {\text {tr}}[\varDelta _-]\ , \end{aligned}$$

where the first optimization ranges over Hermitian *Z* operators and where the second over positive semi-definite operators $\varDelta _\pm$. For any two states $\rho ,\sigma$ (one can even be sub-normalized), the purified distance and the trace distance are related via

2 $$\begin{aligned} D(\rho ,\sigma ) \leqslant P(\rho ,\sigma ) \leqslant \sqrt{2D(\rho ,\sigma )}\ . \end{aligned}$$

Similarly, we may define a distance measure for channels: For two completely positive, trace non-increasing maps ${\mathscr {T}}_{X\rightarrow X'}$ and ${\mathscr {T}}'_{X\rightarrow X'}$, the diamond norm distance is defined as

3 $$\begin{aligned} \frac{1}{2} \left\Vert {{\mathscr {T}}_{X\rightarrow X'} - {\mathscr {T}}'_{X\rightarrow X'}}\right\Vert _\diamond = \max _{\sigma _{XR}}D\bigl ({\mathscr {T}}_{X\rightarrow X'}(\sigma _{XR}), {\mathscr {T}}'_{X\rightarrow X'}(\sigma _{XR})\bigr )\ , \end{aligned}$$

where the optimization ranges over all bipartite quantum states over *X* and a reference system $R\simeq X$. The optimization may be restricted to pure states without loss of generality.

### Entropy measures

The von Neumann entropy of a quantum state $\rho$ is ${H}(\rho ) = -{\text {tr}}[\rho \ln \rho ]$. In this work, all entropies are defined in units of nats, using the natural logarithm $\ln (\cdot )$, instead of units of (qu)bits. A number of nats is equal to $\ln (2)$ times the corresponding number of qubits. The conditional von Neumann entropy of a bipartite state $\rho _{AB}$ is given by

4 $$\begin{aligned} {H}(A\,|\,B)_{\rho } = {H}(AB)_{\rho } - {H}(B)_{\rho } = {H}(\rho _{AB}) - {H}(\rho _B)\ . \end{aligned}$$

The quantum relative entropy is defined as

5 $$\begin{aligned} {D}(\rho \,\Vert \,\sigma ) = {\text {tr}}\bigl [\rho \bigl (\ln \rho - \ln \sigma \bigr )\bigr ]\ , \end{aligned}$$

where $\rho$ is a quantum state and where $\sigma$ is any positive semi-definite operator whose support contains the support of $\rho$.

### Schur–Weyl duality

Consider a Hilbert space ${\mathscr {H}}_A$ and $n\in {\mathbb {N}}$. The group $\mathrm {GL}({d_A})\times \mathrm {S}_{n}$ acts naturally on ${\mathscr {H}}_A^{\otimes n}$, where $X\in \mathrm {GL}({d_A})$ acts as $X^{\otimes n}$ and where the permutation group permutes the tensor factors. We follow closely the notation of Refs. [24, 25]. Schur–Weyl tells us that the full Hilbert space decomposes as

6 $$\begin{aligned} {\mathscr {H}}_A \simeq \bigoplus _\lambda \; {\mathscr {V}}_\lambda = \bigoplus _\lambda \; {\mathscr {Q}}_\lambda \otimes {\mathscr {P}}_\lambda \ , \end{aligned}$$

where $\lambda \in {\text {Young}}(n,d)$ are Young diagrams with *n* boxes and (at most) *d* rows, and where ${\mathscr {Q}}_\lambda$, ${\mathscr {P}}_\lambda$ are irreducible representations of $\mathrm {GL}({d_A})$ and $\mathrm {S}_{n}$, respectively. The number of Young diagrams in the decomposition above is at most ${\text {poly}}(n)$, if $d_A$ is kept constant. We write ${\text {poly}}(n)=O({\text {poly}}(n))$ in big O notation for terms whose absolute value is upper bounded by some polynomial $n^c$ for $c\in {\mathbb {N}}$ in the asymptotic limit $n\rightarrow \infty$.

We denote by $\varPi _{A^n}^\lambda$ the projector in ${\mathscr {H}}_A^{\otimes n}$ onto the term labelled by $\lambda$ in the decomposition above. We denote by $q_\lambda (X)$ a representing matrix of $X\in \mathrm {GL}({d_A})$ in the irreducible representation labelled by $\lambda$; the operator $q_\lambda (X)$ lives in ${\mathscr {Q}}_\lambda$. We furthermore introduce the following notation, for any $Y\in {\mathscr {Q}}_\lambda \otimes {\mathscr {P}}_\lambda$,

7 $$\begin{aligned} \;[\;{Y}\;]_{\lambda }\; = \mathbb {1}_{({\mathscr {Q}}_\lambda \otimes {\mathscr {P}}_\lambda ) \rightarrow A^n} \; Y \; \mathbb {1}_{({\mathscr {Q}}_\lambda \otimes {\mathscr {P}}_\lambda ) \leftarrow A^n}^\dagger \ , \end{aligned}$$

which represents the canonical embedding of an operator *Y* on ${\mathscr {Q}}_\lambda \otimes {\mathscr {P}}_\lambda$ into the space ${\mathscr {H}}_A^{\otimes n}$, i.e., mapping *Y* onto the corresponding block in (6). In particular,

8 $$\begin{aligned} \varPi ^\lambda _{A^n} \;[{Y}]_{\lambda }\; \varPi ^\lambda _{A^n} = \;[{Y}]_{\lambda }\;\ . \end{aligned}$$

Any operator $X_{A^n}$ acting on the *n* copies which commutes with all the permutations admits a decomposition of the form

9 $$\begin{aligned} X_{A^n} = \sum _\lambda \;[{ X_\lambda \otimes \mathbb {1}_{{\mathscr {P}}_\lambda } }]_{\lambda }\; \end{aligned}$$

for some set of operators $X_\lambda \in {\mathscr {Q}}_\lambda$. In particular, $[X_{A^n}, \varPi ^\lambda _{A^n}] = 0$. We can make this more precise for i.i.d. states. For any $X\in \mathrm {GL}({d_A})$, we have that

10 $$\begin{aligned}&{[}\varPi _{A^n}^{\lambda }, X^{\otimes n}] = 0 \end{aligned}$$

11 $$\begin{aligned}&X^{\otimes n} = \sum _\lambda \;[{ q_\lambda (X)\otimes \mathbb {1}_{{\mathscr {P}}_\lambda } }]_{\lambda }\; \ . \end{aligned}$$

For a given $\lambda \in {\text {Young}}(n,d)$, it is often useful to consider the corresponding normalized probability distribution $\lambda /n = (\lambda _i/n)_i$. The entropy of this distribution is given by

12 $$\begin{aligned} {\bar{H}}(\lambda ) = H(\lambda /n) = -\sum _i \frac{\lambda _i}{n}\ln \frac{\lambda _i}{n}\ , \end{aligned}$$

where $\lambda _i$ is the number of boxes in the *i*-th row of the diagram.

If we have *n* copies of a bipartite system ${\mathscr {H}}_A\otimes {\mathscr {H}}_B$, then we may Schur–Weyl decompose ${\mathscr {H}}_A^{\otimes n}$, ${\mathscr {H}}_B^{\otimes n}$ and $({\mathscr {H}}_A\otimes {\mathscr {H}}_B)^{\otimes n}$ under the respective actions of $\mathrm {GL}({d_A})\times \mathrm {S}_{n}$, $\mathrm {GL}({d_B})\times \mathrm {S}_{n}$ and $\mathrm {GL}({d_Ad_B})\times \mathrm {S}_{n}$. A useful property we will need here is that the projectors onto the respective Schur–Weyl blocks commute between these decompositions.

#### Lemma 2.1

Consider two spaces ${\mathscr {H}}_A,{\mathscr {H}}_B$ and let $\varPi ^\lambda _{A^nB^n}$ and $\varPi ^{\lambda '}_{A^n}$ be the projectors onto Schur–Weyl blocks of ${\mathscr {H}}_{AB}^{\otimes n}$ and ${\mathscr {H}}_{A}^{\otimes n}$, respectively, with $\lambda \in {\text {Young}}(d_A d_B,n)$ and $\lambda '\in {\text {Young}}(d_A,n)$. Then, we have

13 $$\begin{aligned} {[} \varPi ^\lambda _{A^nB^n} , \varPi ^{\lambda '}_{A^n} \otimes \mathbb {1}_{B^n} ] = 0\ . \end{aligned}$$

#### Proof

$\varPi ^{\lambda '}_{A^n}\otimes \mathbb {1}_{B^n}$ is invariant under the action of $S_n$ permuting the copies of $A\otimes B$, and so it admits a decomposition of the form (9) and commutes with $\varPi _{A^nB^n}^\lambda$. $\square$

The following is another lemma about how much overlap Schur–Weyl blocks have on a bipartite system versus on one of the two systems. This lemma forms the basis of our universal typical subspace.

#### Lemma 2.2

Consider $n\in {\mathbb {N}}$ copies of a bipartite system ${\mathscr {H}}_A\otimes {\mathscr {H}}_B$. Then, for any $\lambda \in {\text {Young}}(d_A d_B,n)$ and $\lambda '\in {\text {Young}}(d_B,n)$, we have

14 $$\begin{aligned} \varPi ^{\lambda '}_{B^n}\,{\text {tr}}_{A^n}\bigl [\varPi ^\lambda _{A^nB^n}\bigr ]\,\varPi ^{\lambda '}_{B^n} \leqslant {\text {poly}}(n)\, {e}^{n({\bar{H}}(\lambda )-{\bar{H}}(\lambda '))} \, \varPi ^{\lambda '}_{B^n} \end{aligned}$$

noting that $$[\mathbb {1}_{A^n}\otimes \varPi ^{\lambda '}_{B^n}, \varPi ^\lambda _{A^nB^n}]=0$$.

The proof is provided in “Appendix A”.

### Estimating entropy

Measuring the Young diagram $\lambda$—that is, performing the projective measurement with operators $\{ \varPi _{A^n}^\lambda \}_\lambda$—yields a good estimation of the spectrum of a state $\rho _A$ when given $\rho _A^{\otimes n}$ [25]. An estimate for the entropy of $\rho$ is thus obtained by calculating the entropy $H(\lambda /n)$ corresponding to the probability distribution $\lambda /n$.

#### Proposition 2.1

(Spectrum and entropy estimation [22, 24, 25]). Consider $n\in {\mathbb {N}}$ copies of a system ${\mathscr {H}}_A$. Then, the family of projectors $\{ \varPi ^\lambda _{A^n} \}_\lambda$ given by Schur–Weyl duality forms a POVM obeying the following property: For any $\delta >0$, there exists an $\eta >0$ such that for any state $\rho _A$, we have

15 $$\begin{aligned} {\text {tr}} \left[ \left( \sum _{\lambda :~{\bar{H}}(\lambda ) \in [{H}(\rho )\pm \delta ] } \varPi _{A^n}^\lambda \right) \rho _A^{\otimes n} \right] \geqslant 1 - {\text {poly}}(n)\exp \left( - n\eta \right) \ . \end{aligned}$$

The proof is provided in “Appendix A”.

### Estimating energy

#### Proposition 2.2

Consider any observable $H_A$ on ${\mathscr {H}}_A$ and write $\varGamma _A = {e}^{-H_A}$. Then, the set of projectors $\left\{ R_{A^n}^k\right\}$ onto the eigenspaces of $\varGamma _A^{\otimes n}$ forms a POVM satisfying the following properties:

(i) There are at most ${\text {poly}}(n)$ POVM elements, with the label *k* running over a set $k\in {\mathscr {K}}_{n}(H_A)\subset {\mathbb {R}}$;
(ii) We have $[R_{A^n}^k, \varGamma _A^{\otimes n}] = 0$ and ${e}^{-nk}\, R_{A^n}^k = R_{A^n}^k\,\varGamma _A^{\otimes n}$;
(iii) For any $\delta >0$ and for any state $\rho _A$,
  16 $$\begin{aligned} {\text {tr}} \left[ R_{A^n}^{\approx _\delta {\text {tr}}[\rho _A H_A]} \rho _A^{\otimes n} \right] \geqslant 1 - 2 {e}^{-n\eta } \text { with } \eta = \delta ^{2}/(2\Vert H_{A}\Vert _{\infty }^{2}), \end{aligned}$$
  and where for any $h\in {\mathbb {R}}$ we define
  17 $$\begin{aligned} R_{A^n}^{\approx _\delta h} = \sum _{k\in {\mathscr {K}}_{n}(H_A) \;:\; |{k - h}|\leqslant \delta } R_{A^n}^k. \end{aligned}$$
(iv) For any $h\in {\mathbb {R}}$, we have
  18 $$\begin{aligned} {e}^{-n(k+\delta )}R_{A^n}^{\approx _\delta h} \leqslant R_{A^n}^{\approx _\delta h} \, \varGamma _A^{\otimes n} \leqslant {e}^{-n(k-\delta )}R_{A^n}^{\approx _\delta h}\ . \end{aligned}$$

The proof is provided in “Appendix A”.

### Post-selection technique

The post-selection technique is useful for bounding the diamond norm of a candidate smoothed channel to a target ideal i.i.d. channel.

#### Theorem 2.1

(Post-selection technique [18]). Let $X,X'$ be quantum systems, ${\mathscr {E}}_{X\rightarrow X'}$ be a completely positive, trace-preserving map, and ${\mathscr {T}}_{X^n\rightarrow X^{\prime n}}$ be a completely positive, trace non-increasing map. Furthermore, let ${\bar{R}}\simeq X$,

19 $$\begin{aligned} \zeta _{X^n} = {\text {tr}}_{{\bar{R}}^n} \left[ \int d\phi _{X{\bar{R}}}\,|{\phi }\rangle \langle {\phi }|_{X{\bar{R}}}^{\otimes n} \right] = \int d\sigma _X\,\sigma _X^{\otimes n}\ , \end{aligned}$$

where $d\phi _{X{\bar{R}}}$ denotes the Haar-induced measure on the pure states on $X\otimes {\bar{R}}$, and $d\sigma _X$ its induced measure on *X* after partial trace, and let $|{\zeta }\rangle _{X^nR}$ be a purification of $\zeta _{X^n}$. Then, we have

20 $$\begin{aligned} \frac{1}{2}\Vert {{\mathscr {T}} - {\mathscr {E}}^{\otimes n}}\Vert _{\diamond } \leqslant {\text {poly}}(n)\, D\bigl ({\mathscr {T}}(\zeta _{X^nR}), {\mathscr {E}}^{\otimes n}(\zeta _{X^nR})\bigr )\ . \end{aligned}$$

Moreover, for all $n\in {\mathbb {N}}$ there exists a set $\left\{ |{\phi _i}\rangle _{X{\bar{R}}} \right\}$ of at most ${\text {poly}}(n)$ states, and a probability distribution $\left\{ p_i \right\}$, providing a purification of $\zeta _{X^n}$ as

21 $$\begin{aligned} |{\zeta }\rangle _{X^n{\bar{R}}^nR'} = \sum _i \sqrt{p_i}\, |{\phi _i}\rangle _{X{\bar{R}}}^{\otimes n}\otimes |{i}\rangle _{R'} \end{aligned}$$

with a register $R'$ of size ${\text {poly}}(n)$.

The first part of the theorem is [18, Eq. (4)] and the second part is, e.g., found as [23, Cor. D.6]. The following proposition shows that a given channel is close to an i.i.d. channel, if it behaves as expected on all i.i.d. states with exponentially good accuracy.

#### Proposition 2.3

For three systems $X,X',E$, let $V_{X\rightarrow X'E}$ be an isometry and $W_{X^n\rightarrow X'^nE^n}$ be an isometry which commutes with the permutations of the *n* systems. Furthermore, assume that there exists $\eta >0$ independent of *n* such that for all pure states $|{\sigma }\rangle \langle {\sigma }|_{XR_X}$ with a reference system $R_X\simeq X$, we have

22 $$\begin{aligned} {\text {Re}} \left\{ \langle {\sigma }|_{XR_X}^{\otimes n} (V^\dagger _{X\leftarrow X'E})^{\otimes n} \, W_{X^n\rightarrow X'^nE^n} \, |{\sigma }\rangle _{XR_X}^{\otimes n} \right\} \geqslant 1 - {\text {poly}}(n) \exp (-n\eta )\ . \end{aligned}$$

For ${\mathscr {E}}_{X\rightarrow X'}(\cdot ) = {\text {tr}}_E\bigl [V_{X\rightarrow X'E}\,(\cdot )\,V^\dagger \bigr ]$ and ${\mathscr {T}}_{X^n\rightarrow X'^n}(\cdot ) ={\text {tr}}_{E^n}\bigl [W_{X^n\rightarrow {}X'^nE^n}\,(\cdot )\,W^\dagger \bigr ]$ we then have

23 $$\begin{aligned} \frac{1}{2}\bigl \Vert { {\mathscr {T}}_{X^n\rightarrow X'^n} - {\mathscr {E}}_{X\rightarrow X'}^{\otimes n} }\bigr \Vert _\diamond \leqslant {\text {poly}}(n) \exp (-n\eta /2)\ . \end{aligned}$$

The proof is provided in “Appendix A”.

## Resource Theory of Thermodynamics

### Gibbs-preserving maps

We consider the framework of Ref. [16], where for each system *S* considered a positive semi-definite operator $\varGamma _S\geqslant 0$ is associated. A trace non-increasing, completely positive map $\varPhi _{A\rightarrow B}$ is allowed for free if it satisfies $\varPhi _{A\rightarrow B}(\varGamma _A) \leqslant \Gamma _B$. In the case of a system *S* with Hamiltonian $H_S$, and in the presence of a single heat bath at inverse temperature $\beta$, the relevant thermodynamic framework is given by setting $\varGamma _S = {e}^{-\beta H_S}$. In the remainder of this paper, when using the present framework, it is convenient to work with the $\varGamma$ operators on an abstract level. The results then also apply to situations where several different thermodynamic baths are considered, or in more general settings where a specific operator needs to be conserved by the spontaneous evolution of the system [16].

The resources required to enable non-free operations are counted using an explicit system that provides these resources, such as an *information battery*. An information battery is a large register *W* whose associated operator $\varGamma _W$ is simply $\varGamma _W=\mathbb {1}_W$ (i.e., $H_W = 0$). The information battery is required to be in a state of the special form $\tau _W^m = P_W^m/{\text {tr}}[P_W^m]$ where $P_W^m$ is a projector of rank ${e}^{m}$. That is, $\tau _W^m$ has uniform eigenvalues over a given rank ${e}^{m}$. We denote the *charge* or *resource value* of a battery state $\tau _W^m$ by $w(\tau _W^m) = \ln (d) - m$, where *d* is the dimension of the information battery. The value $w(\tau )$ measures the amount of purity present in the state $\tau$, which is the basic resource required to implement maps that are not already Gibbs-preserving maps. We choose to measure $w(\tau )$ in units of number of pure nats, equal to $\ln (2)$ times a number of pure qubits. A Gibbs-preserving map that acts jointly on a system and an information battery, and which maps the input battery state $\tau$ to the output battery state $\tau '$, is deemed to *consume an amount of work* $w = w(\tau ) - w(\tau ')$.

The resources can be counted in terms of thermodynamic work in units of energy if we are given a heat bath at inverse temperature *T*. Recall that a pure qubit can be converted to $kT\ln (2)$ work using a Szilárd engine, where *k* is Boltzmann’s constant [29]. By counting purity in nats instead of qubits, we get rid of the $\ln (2)$ factor: A number $\lambda$ of pure nats can be converted into $\lambda \,kT$ thermodynamic work using a Szilárd-type engine. We count work exclusively in equivalent of pure nats, for simplicity, as opposed to units of energy. The two are directly related by a factor $\beta ^{-1}=kT$. Furthermore, this eliminates the factor $\beta$ from otherwise essentially information-theoretic expressions, and our theorems thus directly apply to cases where $\varGamma _X,\varGamma _{X'}$ are any abstract positive semi-definite operators which are not necessarily defined via a Hamiltonian.

Let $\varPhi _{XW\rightarrow X'W}$ be a Gibbs-preserving map acting on an information battery *W*, and let $\tau _W^{m}$, $\tau _W^{m'}$ be two information battery states. An implementation running the operation $\varPhi _{XW\rightarrow X'W}$ with the given input and output battery states is tasked to (a) make available the input battery state, (b) apply the operation $\varPhi _{XW\rightarrow X'W}$, and (c) check that the output battery state is appropriate (e.g., for possible future re-use). For the verification in Point (c) it is sufficient to measure the two-outcome POVM $\{ P_W^{m'}, \mathbb {1}-P_W^{m'} \}$; as long as the first outcome is observed, it is always possible to bring the state to $\tau _W^{m'}$ by applying a completely thermalizing operation on the support of $P_W^{m'}$ (here, this is a completely randomizing or completely symmetrizing operation). In the constructions presented in the present paper, we allow this verification measurement to fail with a small fixed probability $\epsilon >0$.

A convenient mathematical object to characterize what the operation does on the system is the following. The *effective work process* ${\mathscr {T}}_{X\rightarrow X'F}$ associated with $\varPhi _{XW\rightarrow X'W}$ and $(\tau _W^m,\tau _W^{m'})$ is the trace non-increasing map defined as

24 $$\begin{aligned} {\mathscr {T}}_{X\rightarrow X'}(\cdot )&= {\text {tr}}_W\left[ P_W^{m'}\; \varPhi _{XW\rightarrow X'W}\bigl ( (\cdot ) \otimes \tau _W^m \bigr ) \right] \ . \end{aligned}$$

The question of implementing a process ${\mathscr {E}}$ becomes the issue of finding a Gibbs-preserving map along with battery states such that the associated effective work process is close to ${\mathscr {E}}$. Specifically, if $\Vert { {\mathscr {T}}_{X\rightarrow X'} - {\mathscr {E}}_{X\rightarrow X'}}\Vert _\diamond \leqslant \epsilon$, then we can assert that the failure probability in Point (c) above is bounded by $\epsilon$ for all possible inputs on *X*; the operation therefore implements ${\mathscr {E}}_{X\rightarrow X'}$ accurately with high success probability.

A useful characterization of which processes can be implemented using an information battery is given by the following proposition.

#### Proposition 3.1

( [16, Proposition I]). Let $\varGamma _X,\varGamma _{X'}\geqslant 0$, ${\mathscr {T}}_{X\rightarrow X'}$ be a completely positive, trace non-increasing map, and $w\in {\mathbb {R}}$. Then, the following are equivalent:

(i) We have $$ {\mathscr {T}}_{X\rightarrow X'}(\varGamma _X) \leqslant {e}^{w}\, \varGamma _{X'} $$;
(ii) For all $\delta >0$ there exists an information battery *W* and two battery states $\tau _W,\tau '_W$ such that $w(\tau _W) - w(\tau '_W) \leqslant w+\delta$, and there exists a Gibbs-preserving map $\varPhi _{XW\rightarrow X'W}$ with ${\mathscr {T}}_{X\rightarrow X'}$ the effective work process associated with $\varPhi _{XW\rightarrow X'W}$ and $(\tau _W, \tau '_W)$.

Therefore, to show that one can implement ${\mathscr {E}}_{X\rightarrow X'}$ with Gibbs-preserving maps while expending work *w*, it suffices to exhibit a map ${\mathscr {T}}_{X\rightarrow X'}$ that is $\epsilon$-close to ${\mathscr {E}}_{X\rightarrow X'}$ in diamond distance and that satisfies ${\mathscr {T}}_{X\rightarrow X'}(\varGamma _X)\leqslant {e}^{w}\varGamma _{X'}$. From the proof in [16] we know in Point (ii) above that *W*, $\tau _W \equiv \tau _W^{m}$ and $\tau _W' \equiv \tau _W^{m'}$ can be chosen freely as long as $m' - m = w(\tau _W) - w(\tau '_W) \geqslant w$ and that the corresponding Gibbs-preserving map is given by

25 $$\begin{aligned} \varPhi _{XW\rightarrow X'W}(\cdot )&= {\mathscr {T}}_{X\rightarrow X'}\bigl [ {\text {tr}}_W\bigl (P_W^m\, (\cdot )\bigr ) \bigr ]\,\otimes \tau _W^{m'}\ . \end{aligned}$$

In Ref. [16], the resource cost *w* of implementing a process ${\mathscr {E}}_{X\rightarrow X'}$ (any completely positive, trace-preserving map) up to an accuracy $\epsilon \geqslant 0$ in terms of proximity of the process matrix given a fixed input state $\sigma _X$, counted in pure nats, was shown to be given by the *coherent relative entropy*

26 $$\begin{aligned} w = - {\hat{D}}_{X\rightarrow X'}^{\epsilon }({\mathscr {E}}_{X\rightarrow X'}(\sigma _{XR_X})\,\Vert \,\Gamma _X,\,\Gamma _{X'}) = \ln \min _{\begin{array}{c} {\mathscr {T}}(\Gamma _X)\leqslant \alpha \Gamma _{X'}\\ P({\mathscr {T}}(\sigma _{XR_X}), {\mathscr {E}}(\sigma _{XR_X})) \leqslant \epsilon \end{array}} \alpha \ , \end{aligned}$$

where $\sigma _{XR_X}$ is the purification of $\sigma _X$ on a system $R_X\simeq X$ given by $|{\sigma }\rangle _{XR} = \sigma _X^{1/2}\,|{\varPhi }\rangle _{X:}{R_X}$, and where the optimization ranges over completely positive, trace non-increasing maps ${\mathscr {T}}_{X\rightarrow X'}$. The coherent relative entropy enjoys a collection of properties in relation to the conditional min- and max-entropy, and to the min- and max-relative entropy. It satisfies the following asymptotic equipartition property: For a completely positive, trace-preserving map ${\mathscr {E}}_{X\rightarrow X'}$ and quantum state $\sigma _X$ we have for $0<\epsilon <1$ that

27 $$\begin{aligned} \lim _{n\rightarrow \infty } \frac{1}{n} {\hat{D}}_{X^n\rightarrow X'^n}^{\epsilon } \bigl ({\mathscr {E}}_{X\rightarrow X'}^{\otimes n}(\sigma _{XR}^{\otimes n})\, \big \Vert \,\Gamma _X^{\otimes n},\,\Gamma _{X'}^{\otimes n} \big ) = {D}(\sigma _X\,\Vert \,\Gamma _X) - {D}({\mathscr {E}}(\sigma _X)\,\Vert \,\Gamma _{X'})\ . \end{aligned}$$

### Thermal operations

The framework of Gibbs-sub-preserving maps is technically convenient, but it is unclear whether any Gibbs-sub-preserving operation can be implemented at no work cost using other frameworks. This includes for example thermal operations that might be considered more operational

Here, we consider the alternative framework of *thermal operations* [2, 3, 8]. Each system *S* of interest has an associated Hamiltonian $H_S$ and is not interacting with the other systems. For a given fixed inverse temperature $\beta$, we allow the following operations to be carried out for free:

(i) Apply any unitary operation that commutes with the total Hamiltonian;
(ii) Bring in any ancillary system in its Gibbs state at inverse temperature $\beta$; and
(iii) Discard any system.

The most general transformation a system *S* can undergo under this set of rules is a *thermal operation*. A thermal operations is any process that can be implemented using an additional system *B* with any Hamiltonian $H_B$ and with any unitary $U_{SB}$ satisfying $[U_{SB}, H_S + H_B] = 0$, resulting in the completely positive, trace-preserving map

28 $$\begin{aligned} \varPhi _S(\cdot ) = {\text {tr}}_B\bigl [ U_{SB} \, \bigl ( (\cdot )\otimes \gamma _B \bigr ) \, U_{SB}^\dagger \bigr ]\ , \end{aligned}$$

where $\gamma _B = e^{-\beta H_B} / {\text {tr}}[e^{-\beta H_B}]$ is the Gibbs state of the bath system *B*. Observe that any concatenation of thermal operations is again a thermal operation.

Clearly, any thermal operation $\varPhi _S$ leaves the thermal state $\gamma _S = {e}^{-\beta H_S}/{\text {tr}}[{e}^{-\beta H_S}]$ on *S* invariant. Hence, any lower bound on the work cost of an implementation derived in the framework of Gibbs-preserving maps also applies to thermal operations. We use the same definitions of work and the effective work process for thermal operations as we defined for Gibbs-preserving maps earlier: an information battery is used to account for work, and the effective work process associated with a thermal operation $\varPhi _{XW\rightarrow XW}$ with respect to battery states $(\tau _W^m, \tau _W^{m'})$ is also defined by (24).

When considering only states that commute with the Hamiltonian, a powerful tool to characterize possible state transformations is the notion of thermomajorization [8]. In the fully quantum regime, there is in contrast no known simple mathematical characterization of the work required to implement a quantum process with thermal operations. In fact, because thermal operations are time-covariant, it is impossible to implement processes that are not time-covariant, even if the latter might admit an implementation with a Gibbs-preserving map [6].

We will later use a primitive that transforms a thermal state into a pure energy eigenstate. The next statement follows directly from [8, Eq. (8) and Suppl. Note 4].

#### Proposition 3.2

Let $\gamma _X = {e}^{-\beta H_X}/{\text {tr}}[{e}^{-\beta H_X}]$ be the thermal state on a system *X* with Hamiltonian $H_X$, and let $|{E}\rangle _X$ be a pure energy eigenstate of $H_X$. There exists a thermal operation $\varPhi _{XW}$ on an information battery with battery states $(\tau _W, \tau _W')$ such that $\varPhi _{XW}\bigl (\gamma _X\otimes \tau _W\bigr ) = |{E}\rangle \langle {E}|_X\otimes \tau '_W$ and such that $w(\tau _W)-w(\tau _W')$ can be chosen arbitrarily close to $\beta E + \ln {\text {tr}}[{e}^{-\beta H_X}]$.

## Thermodynamic Capacity

### Definition

Let $X,X'$ be quantum systems, ${\mathscr {E}}_{X\rightarrow X'}$ be a quantum process, and ${\epsilon >0}$. We seek a free thermodynamic operation (either a thermal operation or a Gibbs preserving map) $\varPhi _{X^nW\rightarrow X'^nW}$ that acts on $X^{\otimes n}$ and a battery *W*, with output on $X'^{\otimes n}$ and *W*, as well as information battery states $\tau _W^{\mathrm {(i)}}$ and $\tau _W^{\mathrm {(f)}}$, such that:

(i) The effective work process ${\mathscr {T}}_{X^n\rightarrow X'^n}$ of $\varPhi _{X^nW\rightarrow X'^nW}$ with respect to $\left( \tau _W^{(\mathrm {i})},\tau _W^{(\mathrm {f})}\right)$ is $\epsilon$-close in diamond distance to ${\mathscr {E}}_{X\rightarrow X'}^{\otimes n}$;
(ii) We seek to minimize the work consumption per copy *w* given by
  29 $$\begin{aligned} w = \frac{1}{n}\left[ w\left( \tau _W^{(\mathrm {i})}\right) - w\left( \tau _W^{(\mathrm {f})}\right) \right] \ . \end{aligned}$$

Our main result is a collection of three independent constructions of such implementations in different regimes, using either Gibbs-preserving maps or thermal operations. In each case, the amount of work consumed per copy is given by a quantity which we call the *thermodynamic capacity* of the process, and which turns out to be the minimal work cost an implementation satisfying the above conditions can achieve. The thermodynamic capacity of a completely positive, trace-preserving map ${\mathscr {E}}_{X\rightarrow X'}$ relative to operators $\Gamma _X, \Gamma _{X'} > 0$ is defined as

30 $$\begin{aligned} T({\mathscr {E}}) = \sup _{\sigma _X} \, \Bigl \{ {D}({\mathscr {E}}_{X\rightarrow X'}(\sigma _X)\,\Vert \,\Gamma _{X'}) - {D}(\sigma _X\,\Vert \,\Gamma _X) \Bigr \}\ . \end{aligned}$$

In a fully thermodynamic context where $\Gamma _X = {e}^{-\beta H_X}$ and $\Gamma _{X'} = {e}^{-\beta H'_{X'}}$, one can choose to express the thermodynamic capacity in units of energy rather than in nats, in which case a pre-factor $\beta ^{-1}$ may be included in the definition above such that the thermodynamic capacity is a difference of free energies

31 $$\begin{aligned} T({\mathscr {E}}) = \sup _{\sigma } \Bigl \{ F_{H'}({\mathscr {E}}(\sigma )) - F_H(\sigma ) \Bigr \} \quad \text {with}\quad F_H(\rho ) = \beta ^{-1} {D}(\rho \,\Vert \,{e}^{-\beta H})\ . \end{aligned}$$

*Construction for trivial Hamiltonians* First, in Sect. 5 we consider the special case where $\Gamma _X=\mathbb {1}_X$ and $\Gamma _{X'}=\mathbb {1}_{X'}$ corresponding to trivial Hamiltonians and show that simple considerations based on properties of known entropy measures guarantee the existence of a universal implementation of ${\mathscr {E}}^{\otimes n}$ with either thermal operations or Gibbs-preserving maps.

*Construction using Gibbs-preserving maps* Second, in Sect. 6 we consider the case of general $\Gamma _X,\Gamma _{X'}$ and we construct a universal implementation of ${\mathscr {E}}_{X\rightarrow X'}^{\otimes n}$ with Gibbs-preserving maps, based on new typicality considerations.

*Construction using thermal operations* Third, for arbitrary Hamiltonians we construct in Sect. 7 a universal implementation of ${\mathscr {E}}_{X\rightarrow X'}^{\otimes n}$ with thermal operations, assuming that ${\mathscr {E}}$ is time-covariant, i.e., that it commutes with the time evolution operation.

### Properties

The thermodynamic capacity is a convex optimization program. Namely, the objective function of the optimization in (30) can be written as

32 $$\begin{aligned}&{D}({\mathscr {E}}_{X\rightarrow X'}(\sigma _X)\,\Vert \,\varGamma _{X'}) - {D}(\sigma _X\,\Vert \,\varGamma _X) \nonumber \\&\quad = -{H}({\mathscr {E}}_{X\rightarrow X'}(\sigma _X)) + {H}(\sigma _X) - {\text {tr}} \left[ {\mathscr {E}}_{X\rightarrow X'}(\sigma _X)\,\ln \varGamma _{X'} \right] + {\text {tr}} \left[ \sigma _X\,\ln \varGamma _{X} \right] \nonumber \\&\quad = {H}(E\,|\,X')_{\rho } - {\text {tr}} \left[ {\mathscr {E}}_{X\rightarrow X'}(\sigma _X)\,\ln \varGamma _{X'} \right] + {\text {tr}} \left[ \sigma _X\,\ln \varGamma _{X} \right] \ , \end{aligned}$$

where we defined the state $\rho _{EX'} = V_{X\rightarrow X'E} \sigma _X V^\dagger$ using a Stinespring dilation $V_{X\rightarrow X'E}$ of ${\mathscr {E}}_{X\rightarrow X'}$ into an environment system *E*, satisfying ${\mathscr {E}}_{X\rightarrow X'}(\cdot ) = {\text {tr}}_E \left[ V\,(\cdot )\,V^\dagger \right]$. The conditional entropy is concave in the quantum state as ${H}(E\,|\,X')_{\rho } = { -{D}(\rho _{EX'}\,\Vert \,\mathbb {1}_E\otimes \rho _{X'}) }$ and the quantum relative entropy is jointly convex. The other terms in (32) are linear. Hence, the optimization (30) is a convex optimization that can be carried out efficiently for small system sizes [30]. Indeed, we have successfully computed the thermodynamic capacity of simple example quantum channels acting on few qubits with Python code, using the QuTip framework [31, 32] and the CVXOPT optimization software [33] (see also [34] for a direct algorithm).

The thermodynamic capacity is additive [21]. As a consequence of this property, it is not necessary to include a stabilization over a reference system in the definition of the thermodynamic capacity. That is, had we optimized over bipartite states $\sigma _{XR}$ with a reference system *R* for any $\Gamma _R$, on which the process acts as the identity process, we would be effectively computing $T({\mathscr {E}}\otimes {\mathrm {id}}_{R})$. However, additivity implies that $T({\mathscr {E}}\otimes {\mathrm {id}}_{R}) = T({\mathscr {E}})$.

#### Proposition 4.1

(Additivity of thermodynamic capacity [21]). For $\varGamma _{X},\varGamma _{X'},\varGamma _Z, \varGamma _{Z'} > 0$ and quantum channels ${\mathscr {E}}_{X\rightarrow X'}$, ${\mathscr {F}}_{Z\rightarrow Z'}$ we have

33 $$\begin{aligned} T({\mathscr {E}}\otimes {\mathscr {F}}) = T({\mathscr {E}})+T({\mathscr {F}})\ . \end{aligned}$$

For completeness we provide an independent proof of additivity, to ensure validity in the general setting of abstract $\varGamma$ operators.

#### Proof

Let $\sigma _X,\tau _Z$ be states achieving the thermodynamic capacity of $T({\mathscr {E}})$ and $T({\mathscr {F}})$, respectively. Then, $\sigma _X\otimes \tau _Z$ is a candidate for $T({\mathscr {E}}\otimes {\mathscr {F}})$, yielding

34 $$\begin{aligned} T({\mathscr {E}}\otimes {\mathscr {F}})&\geqslant {D} \bigl ({\mathscr {E}}(\sigma )\otimes {\mathscr {F}}(\tau )\, \big \Vert \,\varGamma _{X'}\otimes \varGamma _{Z'} \big ) - {D} \bigl (\sigma \otimes \tau \, \big \Vert \,\varGamma _{X}\otimes \varGamma _{Z} \big ) \nonumber \\&= {D}({\mathscr {E}}(\sigma )\,\Vert \,\varGamma _{X'}) - {D}(\sigma \,\Vert \,\varGamma _X) + {D}({\mathscr {F}}(\tau )\,\Vert \,\varGamma _{Z'}) - {D}(\tau \,\Vert \,\varGamma _Z) \nonumber \\&= T({\mathscr {E}}) + T({\mathscr {F}})\ . \end{aligned}$$

Now, let $\zeta _{XZ}$ achieve the optimum for $T({\mathscr {E}}\otimes {\mathscr {F}})$. Let $V_{X\rightarrow E_1X'}$, $W_{Z\rightarrow E_2Z'}$ be Stinespring isometries of ${\mathscr {E}}$ and ${\mathscr {F}}$ respectively, such that ${\mathscr {E}}(\cdot ) = {\text {tr}}_{E_1} \left[ V\,(\cdot )\,V^\dagger \right]$ and ${\mathscr {F}}(\cdot ) = {\text {tr}}_{E_2} \left[ W\,(\cdot )\,W^\dagger \right]$. Let $\rho _{E_1E_2X'Z'} = (V\otimes W)\, \zeta \,(V\otimes W)^\dagger$. Then, we have

35 $$\begin{aligned} T({\mathscr {E}}\otimes {\mathscr {F}})&= {D} \bigl (({\mathscr {E}}\otimes {\mathscr {F}})(\zeta )\, \big \Vert \,\varGamma _{X'}\otimes \varGamma _{Z'} \big ) - {D} \bigl (\zeta _{XZ}\, \big \Vert \,\varGamma _X\otimes \varGamma _Z \big ) \nonumber \\&= {H}(E_1E_2\,|\,X'Z')_{\rho } - {\text {tr}} \left[ \rho _{X'Z'} \ln \left( \varGamma _{X'}\otimes \varGamma _{Z'}\right) \right] + {\text {tr}} \left[ \zeta _{XZ} \ln \left( \varGamma _{X}\otimes \varGamma _{Z}\right) \right] \ , \nonumber \\&= {H}(E_1E_2\,|\,X'Z')_{\rho } - {\text {tr}} \left[ \rho _{X'} \ln \left( \varGamma _{X'}\right) \right] - {\text {tr}} \left[ \rho _{Z'}\ln \left( \varGamma _{Z'}\right) \right] \nonumber \\&\quad + {\text {tr}} \left[ \zeta _{X} \ln \left( \varGamma _{X}\right) \right] + {\text {tr}} \left[ \zeta _Z\ln \left( \varGamma _{Z}\right) \right] \end{aligned}$$

since $\ln (A\otimes B) = \ln (A)\otimes \mathbb {1}+ \mathbb {1}\otimes \ln (B)$. Invoking the chain rule of the von Neumann entropy, and then strong sub-additivity of the entropy, we see that ${H}(E_1E_2\,|\,X'Z')_{\rho } = {H}(E_1\,|\,X'Z')_{\rho } + {H}(E_2\,|\,E_1X'Z')_{\rho } \leqslant {H}(E_1\,|\,X')_{\rho } + {H}(E_2\,|\,Z')_{\rho }$. Hence, we have

36 $$\begin{aligned} (35)&\leqslant {H}(E_1\,|\,X')_{\rho } - {\text {tr}} \left[ \rho _{X'}\ln (\varGamma _{X'})\right] + {\text {tr}} \left[ \zeta _X\ln \left( \varGamma _X\right) \right] \nonumber \\&\quad + {H}(E_2\,|\,Z')_{\rho } - {\text {tr}} \left[ \rho _{Z'}\ln (\varGamma _{Z'})\right] + {\text {tr}} \left[ \zeta _Z\ln \left( \varGamma _Z\right) \right] \nonumber \\&\leqslant T({\mathscr {E}}) + T({\mathscr {F}})\ , \end{aligned}$$

where the last inequality holds because the reduced states $\zeta _X, \zeta _Z$ are optimization candidates for $T({\mathscr {E}})$ and $T({\mathscr {F}})$, respectively. $\square$

A special case worth mentioning is when $\varGamma _X=\mathbb {1}_X$, $\varGamma _{X'} = \mathbb {1}_{X'}$, which corresponds to the situation where the Hamiltonians of *X* and $X'$ are trivial. For any quantum channel ${\mathscr {E}}_{X\rightarrow X'}$, let $V_{X\rightarrow X'E}$ be a Stinespring dilation isometry with ${\mathscr {E}}_{X\rightarrow X'} \left( \cdot \right) = {\text {tr}}_E \left[ V\,(\cdot )\,V^\dagger \right]$. Then, we have

37 $$\begin{aligned} T({\mathscr {E}}) = \sup _\sigma \left\{ {H}(\sigma _X) - {H}({\mathscr {E}}(\sigma _X)) \right\} = \sup _\sigma {H}(E\,|\,X')_{V\sigma V^\dagger } \ . \end{aligned}$$

That is, the thermodynamic capacity characterizes by how much the channel is capable of reducing the entropy of its input, or equivalently, how much entropy the channel is capable of dumping into the environment when conditioned on the output. We note that the quantity $-T({\mathscr {E}})$ has previously been studied in the information theory literature as the entropy gain of quantum channels [35–42]. Our work can be seen as giving a precise operational interpretation to this quantity.

### Optimality

Here, we show that any universal implementation that obeys our stated conditions in Sect. 4.1 must necessarily consume an amount of work that is lower bounded by the thermodynamic capacity. That is, any universal implementation that consumes an amount of work equal to the thermodynamic capacity is optimal. This lower bound is simple to prove, because a universal implementation of a process must necessarily be a good implementation for any individual i.i.d. input state, a situation where the optimal work cost is known [16]. Furthermore, any scheme that satisfies the requirements of Sect. 4 at work cost *w* per copy counted with standard battery states of Ref. [16], has an effective process ${\mathscr {T}}_{X^n\rightarrow X'^n}$ on the systems that obeys ${\mathscr {T}}(\varGamma _X^{\otimes n}) \leqslant {e}^{nw}\varGamma _{X'}^{\otimes n}$. This is because any thermal operation is in particular a Gibbs-preserving map, and the work cost is characterized by Proposition 3.1. The following shows that for any such implementation, the work consumed *w* per copy cannot be less than the thermodynamic capacity of the process.

#### Proposition 4.2

Let $\epsilon >0$, $\varGamma _X,\varGamma _{X'} > 0$, ${\mathscr {E}}_{X\rightarrow X'}$ a completely positive, trace-preserving map, and ${\mathscr {T}}_{X^n\rightarrow X'^n}$ a completely positive, trace non-increasing map such that we have $\Vert {{\mathscr {T}} - {\mathscr {E}}^{\otimes n}}\Vert _\diamond /2\leqslant \epsilon$. For $w\in {\mathbb {R}}$ such that ${\mathscr {T}}_{X^n\rightarrow X'^n}(\varGamma _X^{\otimes n}) \leqslant {e}^{nw}\,\varGamma _{X'}^{\otimes n}$, we have in the limit $n\rightarrow \infty$ that $w\geqslant T({\mathscr {E}})$.

#### Proof

Let ${\mathscr {T}}$ with $\frac{1}{2}\Vert {{\mathscr {E}} - {\mathscr {T}}}\Vert _\diamond \leqslant \epsilon$, $\sigma _X$ be a quantum state, and $|{\sigma }\rangle _{XR_X} = \sigma _X^{1/2}\,|{\varPhi }\rangle _{X:R_X}$. Then, by definition of the diamond norm it must hold that $D\bigl ({\mathscr {E}}(\sigma _{XR_X}), {\mathscr {T}}(\sigma _{XR_X})\bigr ) \leqslant \epsilon$, which implies that $P\bigl ({\mathscr {E}}(\sigma _{XR_X}), {\mathscr {T}}(\sigma _{XR_X})\bigr ) \leqslant \sqrt{2\epsilon }$. We have that ${\mathscr {T}}$ is a valid optimization candidate for the definition of the coherent relative entropy and thus

38 $$\begin{aligned} -{\hat{D}}_{X^n\rightarrow X'^n}^{\sqrt{2\epsilon }} \bigl ({\mathscr {E}}_{X\rightarrow X'}^{\otimes n}(\sigma _{XR_X}^{\otimes n})\, \big \Vert \,\varGamma _X^{\otimes n},\,\varGamma _{X'}^{\otimes n} \big ) \leqslant nw\ . \end{aligned}$$

For $n\rightarrow \infty$, we can employ the asymptotic equipartition of the coherent relative entropy (27) to see that

39 $$\begin{aligned} {D}({\mathscr {E}}(\sigma _X)\,\Vert \,\varGamma _{X'}) - {D}(\sigma _X\,\Vert \,\varGamma _X) \leqslant w\ . \end{aligned}$$

Since this inequality holds for all $\sigma _X$, we deduce that $T({\mathscr {E}})\leqslant w$. $\square$

## Construction #1: Trivial Hamiltonians

### Statement and proof sketch

Instead of constructing explicitly an implementation that satisfies the requirements of Sect. 4, one might hope that the implementation could be given implicitly as the solution of a semi-definite program representing an entropy measure. This proof idea was indeed exploited in other contexts in Refs. [23, 43]. Here, we define the one-shot entropy-like quantity

40 $$\begin{aligned} {W}_{X\rightarrow X'}^{\epsilon }({\mathscr {E}}_{X\rightarrow X'}\,\Vert \,\varGamma _X,\,\varGamma _{X'}) = \min _{\begin{array}{c} {\mathscr {T}}(\varGamma _X) \leqslant {e}^{w} \varGamma _{X'}\\ \frac{1}{2} \left\Vert {{\mathscr {T}} - {\mathscr {E}}}\right\Vert _\diamond \leqslant \epsilon \end{array}} w \ , \end{aligned}$$

where ${\mathscr {T}}_{X\rightarrow X'}$ ranges over all trace non-increasing, completely positive maps. The proof strategy would then be to relate this entropy measure to the coherent relative entropy, and to exploit known properties of the latter in the i.i.d. regime to provide an upper bound to the expression

41 $$\begin{aligned} \frac{1}{n}{W}_{X^n\rightarrow X'^n}^{\epsilon }({\mathscr {E}}^{\otimes n}_{X^n\rightarrow X'^n}\,\Vert \,\varGamma _X^{\otimes n},\,\varGamma _{X'}^{\otimes n})\ . \end{aligned}$$

Should this upper bound behave like $T({\mathscr {E}})$ to leading order, then the ${\mathscr {T}}$ equal to the optimal solution to (40) defines an implementation in terms of Gibbs-preserving maps thanks to Proposition 3.1. It turns out that this proof strategy works well in the special case of trivial Hamiltonians, but fails in the general case.

The core technical statement that underlies our Construction #1 is summarized in the following theorem.

#### Theorem 5.1

Let ${\mathscr {E}}_{X\rightarrow X'}$ be a completely positive, trace-preserving map, and $\epsilon >0$. Then we have

42 $$\begin{aligned} \lim _{n\rightarrow \infty } \frac{1}{n} {W}_{X^n\rightarrow X'^n}^{\epsilon }({\mathscr {E}}^{\otimes n}_{X^n\rightarrow X'^n}\,\Vert \,\mathbb {1}_{X^n},\,\mathbb {1}_{X'^n}) = T({\mathscr {E}})\ , \end{aligned}$$

where $$T({\mathscr {E}}) = \max _{\sigma _X}\left\{ {H}(\sigma _X) -{H}({\mathscr {E}}(\sigma _X))\right\} $$.

This implementation is constructed by taking the implicit optimal solution ${\mathscr {T}}_{X^n\rightarrow X'^n}$ in the semi-definite program (40) for $\frac{1}{n} {W}_{X^n\rightarrow X'^n}^{\epsilon }({\mathscr {E}}_{X\rightarrow X'}^{\otimes n}\,\Vert \,\mathbb {1}_{X^n},\,\mathbb {1}_{X'^n})$, and using Proposition 3.1 to construct an associated Gibbs-preserving map acting on battery states via (25). In summary, for any $\delta '>0$, for *n* large enough and choosing any $m,m'$ such that $m-m' \leqslant nT({\mathscr {E}}) + \delta '$, the full implementation map in terms of ${\mathscr {T}}_{X^n\rightarrow X'^n}$ becomes

43 $$\begin{aligned} \varPhi _{X^nW\rightarrow X'^nW}(\cdot )&= {\mathscr {T}}_{X^n\rightarrow X'^n}\bigl ( {\text {tr}}_W[ P_W^m (\cdot ) ] \bigr ) \otimes \tau _W^{m'}\ . \end{aligned}$$

We emphasise that Theorem 5.1 exactly covers the entropy gain of quantum channels as studied in [35–42].

#### Proof

(Theorem 5.1) By using the post-selection technique (Theorem 2.1) and recalling that the fixed-input state case is given by the coherent relative entropy, we find

44 $$\begin{aligned} {W}_{X^n\rightarrow X'^n}^{\epsilon } \bigl ({\mathscr {E}}^{\otimes n}_{X\rightarrow X'}\, \big \Vert \,\mathbb {1}_{X^n},\,\mathbb {1}_{X'^n} \big ) \leqslant - {\hat{D}}_{X^n\rightarrow X'^n}^{\epsilon /\!{\text {poly}}(n)} \bigl ({\mathscr {E}}^{\otimes n}_{X\rightarrow X'}(\zeta _{X^nR_X^n})\, \big \Vert \,\mathbb {1}_{X^n},\,\mathbb {1}_{X'^n} \big )\ . \end{aligned}$$

In the case of trivial Hamiltonians, the coherent relative entropy reduces to the smooth max-entropy (cf. [16, Props. 28 and 26] and also Ref. [44]). More precisely, we have

45 $$\begin{aligned} {\hat{D}}_{X\rightarrow X'}^{\epsilon } \bigl (\rho _{X'R_{X}}\, \big \Vert \,\mathbb {1}_X,\,\mathbb {1}_{X'} \big ) \geqslant - {H}_{\mathrm {max}}^{c\epsilon ^\alpha }(E\,|\,X')_{\rho } + g(\epsilon )\ , \end{aligned}$$

where $|{\rho }\rangle _{X'R_XE}$ is a pure state, where $c>0$, $0<\alpha <1$, $g(\epsilon )$ are universal and do not depend on the state or the dimensions of the systems, and the smooth max-entropy is defined as

46 $$\begin{aligned} {H}_{\mathrm {max}}^{\epsilon }(E\,|\,X')_{\rho }&= \min _{P({\hat{\rho }}, \rho )\leqslant \epsilon } {H}_{\mathrm {max}}(E\,|\,X')_{{\hat{\rho }}}\ ; \\ {H}_{\mathrm {max}}(E\,|\,X')_{{\hat{\rho }}}&= \max _{0\le \omega _{X'}\le \mathbb {1}} \, \ln \, \bigl \Vert {{\hat{\rho }}_{EX'}^{1/2}\omega _{X'}^{1/2}}\bigr \Vert ^2_1\ . \end{aligned}$$

Thus, we have

47 $$\begin{aligned} (44) \leqslant {H}_{\mathrm {max}}^{\epsilon ^\alpha /\!{\text {poly}}(n)}( E^n \,|\, X'^n )_{\rho } + g(\epsilon )\ , \end{aligned}$$

where $\rho _{X'^nE^n} = V_{X\rightarrow X'E}^{\otimes n} \zeta _{X^n} (V^\dagger )^{\otimes n} = \int d\sigma \, (V\sigma V^\dagger )^{\otimes n}$ and $V_{X\rightarrow X'E}$ is a Stinespring dilation isometry of ${\mathscr {E}}_{X\rightarrow X'}$ as ${\mathscr {E}}_{X\rightarrow X'}(\cdot ) = {\text {tr}}_E \left[ V_{X\rightarrow X'E}\,(\cdot )\, V^\dagger \right]$. At this point we invoke two facts. First, note that the de Finetti state can be written as a mixture of only ${\text {poly}}(n)$ i.i.d. states, instead of a continuous average (Theorem 2.1): There exists a set $\{ \sigma _i \}$ of at most ${\text {poly}}(n)$ states and a distribution $\{ p_i \}$ such that $\zeta _{X^n} = \sum _i p_i \sigma _i^{\otimes n}$. Second, we invoke the property that the conditional max-entropy is quasi-convex up to a penalty term, namely, that the conditional max-entropy of $\sum _i p_i \rho _i$ is less than or equal to the maximum over the set of max-entropies corresponding to each $\rho _i$, plus a term proportional to the logarithm of the number of terms in the sum [45, Lemma 11]. Hence, with $\rho _i = V\,\sigma _i\,V^\dagger$, we get

48 $$\begin{aligned} (48) \leqslant \max _i {H}_{\mathrm {max}}^{\epsilon ^\alpha /\!{\text {poly}}(n)}( E^n \,|\, X'^n )_{\rho _i^{\otimes n}} + \ln ({\text {poly}}(n)) + g(\epsilon ) \ . \end{aligned}$$

Now, we are in business because the max-entropy is evaluated on an i.i.d. state, and we know that it asymptotically goes to the von Neumann entropy in this regime [46]. Also, $\lim _{n\rightarrow \infty } (1/n)\big \{\ln ({\text {poly}}(n)) + g(\epsilon )\big \}=0$ and hence

49 $$\begin{aligned} \lim _{n\rightarrow \infty } \frac{1}{n} {W}_{X^n\rightarrow X'^n}^{\epsilon } \bigl ({\mathscr {E}}^{\otimes n}_{X\rightarrow X'}\, \big \Vert \,\mathbb {1}_{X^n},\,\mathbb {1}_{X'^n} \big )&\leqslant \max _i\, {H}(E\,|\,X')_{\rho _i} \nonumber \\&= \max _i \left\{ {H}(\sigma _i) - {H}({\mathscr {E}}(\sigma _i)) \right\} \nonumber \\&\leqslant \max _\sigma \left\{ {H}(\sigma ) - {H}({\mathscr {E}}(\sigma )) \right\} \nonumber \\&= T({\mathscr {E}}) \end{aligned}$$

noting that $${H}(E\,|\,X') = {H}(EX') - {H}(X') = {H}(X) - {H}(X')$$. $$\square $$

### Challenges for extension to non-trivial Hamiltonians

Naturally, one might ask whether it is possible to extend this proof to the case of non-trivial $\Gamma$ operators. Interestingly, this is not possible, at least not in a naive way. The problem is that we need a quasi-convexity property of the form

50 $$\begin{aligned}&- {\hat{D}}_{X\rightarrow X'}^{\epsilon } \bigl ({\mathscr {E}}_{X\rightarrow X'}(\sigma _{XR_X})\, \big \Vert \,\varGamma _{X},\,\varGamma _{X'} \big ) \nonumber \\&\quad {\mathop {\leqslant }\limits ^{{?}}} \max _i \left( - {\hat{D}}_{X\rightarrow X'}^{\epsilon } \bigl ({\mathscr {E}}_{X\rightarrow X'}(\sigma ^i_{XR_X})\, \big \Vert \,\varGamma _{X},\,\varGamma _{X'} \big ) \right) + \text {(penalty)}\ , \end{aligned}$$

where $\sigma _X = \sum p_i \sigma _X^i$ and $|{\sigma }\rangle _{XR} = \sigma _X^{1/2}\,|{\varPhi }\rangle _{X:R_X}$, $|{\sigma ^i}\rangle _{XR} = (\sigma ^i_X)^{1/2}\,|{\varPhi }\rangle _{X:R_X}$, and where the $\text {(penalty)}$ term scales in a favourable way in *n*, say of order $\ln ({\text {poly}}(M))$ where *M* is the number of terms in the convex decomposition as for the max-entropy. In fact, Eq. (51) is false, as can be shown using an explicit counterexample on a two-level system which we present below. As this example is based on physical reasons, the coherent relative entropy is not even approximately quasi-convex. We note that a priori we cannot rule out a quasi-convexity property that might have a penalty term that depends on properties of the $\Gamma$ operators, yet such a term is likely to scale unfavourably with *n*.

Our example is as follows. Consider a two-level system with a Hamiltonian *H* with energy levels $|{0}\rangle ,|{1}\rangle$ at corresponding energies $E_0=0$ and $E_1>0$. The corresponding $\Gamma$ operator is $\varGamma = g_0|{0}\rangle \langle {0}|+ g_1|{1}\rangle \langle {1}|$ with $g_0 = 1$, $g_1 = {e}^{-\beta E_1}$. Consider the process consisting in erasing the input and creating the output state $|{+}\rangle$, where we define $|{\pm }\rangle = [|{0}\rangle \pm |{1}\rangle ]/\sqrt{2}$. That is, we consider the process ${\mathscr {E}}(\cdot ) = {\text {tr}}[\cdot ]\,|{+}\rangle \langle {+}|$. Suppose the input state is maximally mixed, $\sigma =\mathbb {1}/2$, such that $\rho _{X'R_X} = |{+}\rangle \langle {+}|_{X'}\otimes \mathbb {1}_{R_X}/2$. If $E_0=0$ and $E_1\rightarrow \infty$, then this process requires a lot of work; intuitively, with probability 1/2 we start in the ground state $|{0}\rangle$ and need to prepare the output state $|{+}\rangle$ which has high energy.

For $\epsilon =0$, we can see this because the input state is full rank, hence ${\mathscr {T}}={\mathscr {E}}$; then ${\mathscr {E}}(\varGamma ) = {\text {tr}}[\varGamma ]|{+}\rangle \langle {+}|$ and the smallest $\alpha$ such that ${\mathscr {E}}(\varGamma ) \leqslant \alpha \varGamma$ is given by

51 $$\begin{aligned}&\alpha /{\text {tr}}[\varGamma ] = \bigl \Vert {\varGamma ^{-1/2}|{+}\rangle \langle {+}|\varGamma ^{-1/2}}\bigr \Vert _\infty = \langle {+}\vert {\varGamma ^{-1}}\vert {+}\rangle = (g_0^{-1} + g_1^{-1})/2 \nonumber \\&\quad = (1 + {e}^{\beta E_1})/2 \geqslant {e}^{\beta E_1}/2\ . \end{aligned}$$

Noting that ${\text {tr}}[\varGamma ]\geqslant 1$, we have $\alpha \geqslant {e}^{\beta E_1}/2$, and hence the energy cost of the transformation $\mathbb {1}/2\rightarrow |{+}\rangle$ is

52 $$\begin{aligned} \text {energy cost} = - \beta ^{-1}{\hat{D}}_{X\rightarrow X'}({\mathscr {E}}_{X\rightarrow X'}(\sigma _{XR_X})\,\Vert \,\varGamma ,\,\varGamma ) = \beta ^{-1} \ln \alpha \geqslant E_1 - \beta ^{-1}\ln (2)\ . \end{aligned}$$

Clearly, this work cost can become arbitrarily large if $E_1\rightarrow \infty$. On the other hand, we can perform the transformation $|{+}\rangle \rightarrow |{+}\rangle$ obviously at no work cost; similarly, $|{-}\rangle \rightarrow |{+}\rangle$ can be carried out by letting the system time-evolve under its own Hamiltonian for exactly the time interval required to pick up a relative phase $(-1)$ between the $|{0}\rangle$ and $|{1}\rangle$ states. This also costs no work because it is a unitary operation that commutes with the Hamiltonian. We thus have our counter-example to the quasi-convexity of the coherent relative entropy. The transformation $\mathbb {1}/2\rightarrow |{+}\rangle$ is very hard, but the individual transformations $|{\pm }\rangle \rightarrow |{+}\rangle$ are trivial, noting that $\mathbb {1}/2=(1/2)|{+}\rangle \langle {+}|+ (1/2)|{-}\rangle \langle {-}|$.

We show in “Appendix D” how to make the above claim robust against an accuracy tolerance $\epsilon \ge 0$.

## Construction #2: Gibbs-Preserving Maps

### Statement and proof sketch

Here, we present a general construction of a universal implementation of an i.i.d. process using Gibbs-preserving maps according to the requirements of Sect. 4.1. The idea is to explicitly construct an implementation using a novel notion of quantum typicality. We introduce notions of quantum typicality that apply to quantum processes and universally capture regions of the Hilbert space where the conditional entropy (respectively the relative entropy difference) has a given value. This generalizes existing notions of typical projectors to a quantum typical operator that applies to bipartite states, is relative to a $\Gamma$ operator, and universal.

The main result behind the construction in this section is the following theorem.

#### Theorem 6.1

Let $\varGamma _X,\varGamma _{X'} > 0$, ${\mathscr {E}}_{X\rightarrow X'}$ be a completely positive, trace-preserving map, and $\epsilon >0$. Then, for $\delta >0$ and $n\in {\mathbb {N}}$ large enough there exists a completely positive map ${\mathscr {T}}_{X^n\rightarrow X'^n}$ such that:

(i) ${\mathscr {T}}_{X^n\rightarrow X'^n}$ is trace non-increasing;
i(ii) $$\bigl \Vert {{\mathscr {T}}_{X^n\rightarrow X'^n} - {\mathscr {E}}_{X\rightarrow {}X'}^{\otimes {}n}}\bigr \Vert _\diamond \leqslant \epsilon $$;
(iii) $${\mathscr {T}}_{X^n\rightarrow X'^n}\bigl (\varGamma _X^{\otimes n}\bigr ) \leqslant {e}^{n[T({\mathscr {E}}) + 4\delta + n^{-1}\ln ({\text {poly}}(n))]}\,\varGamma _{X'}^{\otimes n}$$.

Note that we have $n^{-1}\ln ({\text {poly}}(n)) \rightarrow 0$ as $n\rightarrow \infty$, and that we can take $\delta \rightarrow 0$ after taking $n\rightarrow \infty$. Thanks to Proposition 3.1, the mapping ${\mathscr {T}}_{X^n\rightarrow X'^n}$ defines an implementation of the i.i.d. process ${\mathscr {E}}_{X\rightarrow X'}^{\otimes n}$ in terms of Gibbs-preserving maps and a battery, whose work cost rate is given to leading order by the thermodynamic capacity $T({\mathscr {E}})$ after taking $\delta \rightarrow 0$.

As for Construction #1, the full Gibbs-preserving map implementing the required process is assembled in two steps, first constructing the map ${\mathscr {T}}_{X^n\rightarrow X'^n}$ in Theorem 6.1 and then using Proposition 3.1 to obtain the full Gibbs-preserving map. Let $V_{X\rightarrow X'E}$ be a Stinespring dilation isometry of ${\mathscr {E}}_{X\rightarrow X'}$. For $\delta >0$, we introduce a universal conditional and relative typical smoothing operator $M_{E^nX'^n}^{x,\delta }$ (see later Definition 6.1 and Proposition 6.1) with $x = -nT({\mathscr {E}})$ and relative to $\varGamma _{X'E} \equiv V\varGamma _X V^\dagger$ and $\varGamma _{X'}$. The map ${\mathscr {T}}_{X^n\rightarrow X'^n}$ is then constructed as

53 $$\begin{aligned} {\mathscr {T}}_{X^n\rightarrow X'^n}(\cdot )&= {\text {tr}}_{E^n} \left[ M_{E^nX'^n}^{x,\delta } \,V_{X\rightarrow X'E}^{\otimes n}\, (\cdot )\, V_{X\leftarrow X'E}^{\dagger \,\otimes n} M_{E^nX'^n}^{x,\delta \,\dagger } \right] \ . \end{aligned}$$

Finally, we employ Proposition 3.1 to construct an associated Gibbs-preserving map acting on battery states via (25). For any $\delta '>0$, for *n* large enough and choosing any $m,m'$ such that $m-m' \leqslant nT({\mathscr {E}}) + 4\delta + n^{-1}\ln {\text {poly}}(n) + \delta '$, the full implementation map in terms of ${\mathscr {T}}_{X^n\rightarrow X'^n}$ becomes

54 $$\begin{aligned} \varPhi _{X^nW\rightarrow X'^nW}(\cdot )&= {\mathscr {T}}_{X^n\rightarrow X'^n}\bigl ( {\text {tr}}_W[ P_W^m (\cdot ) ] \bigr ) \otimes \tau _W^{m'}\ . \end{aligned}$$

### Construction via universal conditional and relative typicality

The main ingredient of our proof is a notion of a universal conditional and relative typical smoothing operator that enables us to discard events that are very unlikely to appear in the process while accounting for how much they contribute to the overall work cost. This operator is inspired by similar constructions in Refs. [47, 48]. However, in additional to being “relative” as in [47] our smoothing operator is also simultaneously “conditional” and “universal”.

#### Definition 6.1

Let $\Gamma _{AB},\Gamma _B'\geqslant 0$ and $x\in {\mathbb {R}}$. A *universal conditional and relative typical smoothing operator* $M_{A^nB^n}^{x,\delta }$ with parameter $\delta >0$ is an operator on $A^nB^n$ that satisfies the following conditions:

(i) $$\bigl (M^{x,\delta }_{A^nB^n}\bigr )^\dagger \; M^{x,\delta }_{A^nB^n} \leqslant \mathbb {1}$$ ;
(ii) There exists $\xi >0$ independent of *n* with the following property: For any pure state $|{\rho }\rangle _{ABR}$ with $\rho _{AB}$ (respectively $\rho _B$) in the support of $\varGamma _{AB}$ (respectively $\varGamma _B'$) and such that ${D}(\rho _{AB}\,\Vert \,\varGamma _{AB}) - {D}(\rho _B\,\Vert \,\varGamma _B') \geqslant x$, it holds that
  55 $$\begin{aligned} {\text {Re}} \left\{ \langle {\rho }|_{ABR}^{\otimes n} \, M^{x,\delta }_{A^nB^n}\, |{\rho }\rangle _{ABR}^{\otimes n} \right\} \geqslant 1 - {\text {poly}}(n)\exp (-n\xi )\ ; \end{aligned}$$
(iii) $${\text {tr}}_{A^n}\Bigl [M^{x,\delta }_{A^nB^n}\,\varGamma _{AB}^{\otimes n}\, \bigl (M^{x,\delta }_{A^nB^n}\bigr )^\dagger \Bigr ] \leqslant {\text {poly}}(n)\,{e}^{-n(x-4\delta )}\,\varGamma _{B}'^{\otimes n}$$ .

Note that the smoothing operator is defined as a general operator of norm bounded by one, as opposed to the usual definition of typical subspaces or typical projectors. The main reason is that it is not known to us in general if such an object can be chosen to be a projector. By using the real part in Point (ii) above, we ensure that a process that applies the operator $M_{A^nB^n}^{x,\delta }$ preserves coherences when it is applied to a superposition of several states $\{ |{\rho }\rangle _{ABR}^{\otimes n} \}$. This property would not have been ensured if instead, we had merely asserted that $M_{A^nB^n}^{x,\delta }|{\rho }\rangle _{ABR}^{\otimes n}$ and $|{\rho }\rangle _{ABR}^{\otimes n}$ have high absolute value overlap or are close in fidelity. If $M_{A^nB^n}^{x,\delta }$ is a projector then the expression reduces to ${\text {tr}}(M_{A^nB^n}^{x,\delta }\rho )$ as one usually considers for projectors on typical subspaces.

The core technical statement of Construction #2 is to show the existence of a universal conditional and relative smoothing operator, which is as follows.

#### Proposition 6.1

Let $\Gamma _{AB},\Gamma _B'\geqslant 0$, $x\in {\mathbb {R}}$, as well as $n\in {\mathbb {N}}$ and $\delta >0$. There exists a universal conditional and relative typical smoothing operator $M_{A^nB^n}^{x,\delta }$ that is furthermore permutation-invariant. Moreover, if $[\Gamma _{AB}, \mathbb {1}_A\otimes \Gamma _B']=0$, then $M^{x,\delta }_{A^nB^n}$ can be chosen to be a projector satisfying $[M^{x,\delta }_{A^nB^n},\Gamma _{B}'^{\otimes n}] = 0$ and $[M^{x,\delta }_{A^nB^n},\Gamma _{AB}^{\otimes n}] = 0$.

In the following, we present the proof of Theorem 6.1 based on the existence of such the smoothing operator from Proposition 6.1. The more technical proof of Proposition 6.1 is then given in Sect. 6.3.

#### Proof

(Theorem 6.1). Let $V_{X\rightarrow X'E}$ be a Stinespring dilation of ${\mathscr {E}}_{X\rightarrow X'}$ into an environment system $E\simeq X\otimes X'$. For $n\in {\mathbb {N}}$ we need to find a suitable candidate implementation ${\mathscr {T}}_{X^n\rightarrow X'^n}$. Let

56 $$\begin{aligned} x = -\max _{\sigma _X}\Big \{ {D}({\mathscr {E}}(\sigma _X)\,\Vert \,\Gamma _{X'}) - {D}(\sigma _X\,\Vert \,\Gamma _X) \Big \} = -T({\mathscr {E}})\ . \end{aligned}$$

For any $\delta >0$ let $M^{x,\delta }_{E^nX'^n}$ be the operator constructed by Proposition 6.1, with the system *E* playing the role of the system *A*, with $V_{X\rightarrow X'E}\,\Gamma _X\,V_{X\leftarrow X'E}^\dagger$ as $\Gamma _{AB}$ and with $\Gamma _{X'}$ as $\Gamma '_{B}$. Now, define

57 $$\begin{aligned} {\mathscr {T}}_{X^n\rightarrow X'^n}(\cdot ) = {\text {tr}}_{E^n} \left[ M^{x,\delta }_{E^nX'^n} V_{X\rightarrow X'E}^{\otimes n} \; \bigl (\cdot \bigr ) \; \bigl (V^\dagger _{X\leftarrow X'E}\bigr )^{\otimes n} \bigl (M^{x,\delta }_{E^nX'^n}\bigr )^\dagger \right] \end{aligned}$$

noting that ${\mathscr {T}}_{X^n\rightarrow X'^n}$ is trace non-increasing by construction thanks to Property (i) of Definition 6.1.

Let $|{\sigma }\rangle _{XR_X}$ be any pure state, and define $|{\rho }\rangle _{X'ER_X} = V_{X\rightarrow X'E}\,|{\sigma }\rangle _{XR_X}$. By construction, ${D} \bigl (\rho _{EX'}\, \big \Vert \,(V_{X\rightarrow X'E}\Gamma _{X}V^\dagger ) \big ) - {D}(\rho _{X'}\,\Vert \,\Gamma _{X'}) = {D}(\sigma _{X}\,\Vert \,\Gamma _{X}) - {D}({\mathscr {E}}(\sigma _{X})\,\Vert \,\Gamma _{X'}) \geqslant x$. Then Property (ii) of Proposition 6.1 tells us that there exists a $\xi >0$ independent of both $\rho$ and *n* such that

58 $$\begin{aligned} {\text {Re}} \left\{ \langle {\rho }|_{X'ER_X}^{\otimes n}\, M^{x,\delta }_{E^nX'^n}\, |{\rho }\rangle _{X'ER_X}^{\otimes n} \right\} \geqslant 1 - {\text {poly}}(n)\,\exp (-n\xi )\ . \end{aligned}$$

The conditions of Proposition 2.3 are fulfilled, with $W_{X^n\rightarrow X'^nE^n} = M^{x,\delta }_{A^nB^n} \, V_{X\rightarrow X'E}^{\otimes n}$, thanks furthermore to the fact that $M_{E^nX'^n}^{x,\delta }$ is permutation-invariant as guaranteed by Proposition 6.1. Hence, we have

59 $$\begin{aligned} \frac{1}{2}\bigl \Vert { {\mathscr {T}}_{X^n\rightarrow X'^n} - {\mathscr {E}}_{X\rightarrow X'}^{\otimes n} }\bigr \Vert _\diamond \leqslant {\text {poly}}(n)\,\exp (-n\xi /2)\ . \end{aligned}$$

For $n\in {\mathbb {N}}$ large enough this becomes smaller than any fixed $\epsilon >0$. Furthermore, by Property (iii) of Definition 6.1, we have that

60 $$\begin{aligned} {\mathscr {T}}_{X^n\rightarrow X'^n}\bigl (\Gamma _{X}^{\otimes n}\bigr )&= {\text {tr}}_{E^n}\bigl [ M^{x,\delta }_{E^nX'^n} \, \bigl (V_{X\rightarrow X'E} \, \Gamma _X V_{X\leftarrow X'E}^\dagger \bigr )^{\otimes n} (M^{x,\delta }_{E^nX'^n})^\dagger \bigr ] \nonumber \\&\leqslant {\text {poly}}(n)\,{e}^{-n(x-4\delta )}\,\Gamma _{X'}^{\otimes n} \end{aligned}$$

as required. $$\square $$

### Universal conditional and relative typical smoothing operator

We now turn to the proof of Proposition 6.1, giving an explicit construction of a universal conditional and relative typical smoothing operator. As the proof of Proposition 6.1 is quite lengthy, it can be instructive to consider a simpler version of our typical smoothing operator which applies in the case where the Hamiltonians are trivial. We carry out this analysis in “Appendix E”.

#### Proof

(Proposition 6.1). First, we claim that we can assume $\Gamma _{AB}>0$ and $\Gamma _B'>0$ without loss of generality. Indeed, if either operator is not positive definite, then we can first construct the operator ${\widetilde{M}}_{A^nB^n}^{x,\delta }$ associated with modified operators ${\widetilde{\Gamma }}_{AB}>0$ and ${\widetilde{\Gamma }}_B'>0$ where all the zero eigenvalues of $\Gamma _{AB}$ and $\Gamma _B'$ are replaced by some arbitrary fixed strictly positive constant (e.g., one); we can then set $M_{A^nB^n}^{x,\delta } = P^{\Gamma '}_{B^n} {\widetilde{M}}_{A^nB^n}^{x,\delta } P^\Gamma _{A^nB^n}$, where $P^{\Gamma }_{A^nB^n}$ (respectively $P^{\Gamma '}_{B^n}$) is the projector onto the support of $\Gamma _{AB}^{\otimes n}$ (respectively $\Gamma _B'^{\otimes n}$). The operator $M_{A^nB^n}^{x,\delta }$ constructed in this way satisfies all of the required properties. For the remainder of this proof we thus assume that $\Gamma _{AB}>0$ and $\Gamma _B'>0$.

Let $\left\{ R_{A^nB^n}^k\right\}$ be the POVM constructed by Proposition 2.2 for $H_{AB} = -\ln (\Gamma _{AB})$. Similarly, let $\left\{ S_{B^n}^\ell \right\}$ be the corresponding POVM constructed in Proposition 2.2 for $H_{B}' = -\ln (\Gamma '_{B})$. Also, as before, we denote by $\varPi ^\lambda _{A^nB^n}$ and by $\varPi ^{\mu }_{B^n}$ the projectors on the Schur–Weyl blocks labelled by the Young diagrams $\lambda \in {\text {Young}}(d_{A}d_{B},n)$ and $\mu \in {\text {Young}}(d_{B},n)$. Let

61 $$\begin{aligned} M^{x,\delta }_{A^nB^n} = \sum _{\begin{array}{c} k,\ell ,\lambda ,\mu \;:\\ k-{\bar{H}}(\lambda )-\ell +{\bar{H}}(\mu ) \geqslant x - 4\delta \end{array}} S^\ell _{B^n}\,\varPi ^{\mu }_{B^n}\,\varPi ^\lambda _{A^nB^n}\,R^k_{A^nB^n}\ . \end{aligned}$$

Note that $[S^\ell _{B^n}, \varPi ^{\mu }_{B^n}] = 0$ because $S^\ell _{B^n}$ is permutation-invariant, and $[\mathbb {1}_{A^n}\otimes S^\ell _{B^n}, \varPi ^{\lambda }_{A^nB^n}] = 0$ because $\mathbb {1}_{A^n}\otimes S^\ell _{B^n}$ is permutation-invariant. Recall also that $[\mathbb {1}_{A^n}\otimes \varPi ^\mu _{B^n}, \varPi ^{\lambda }_{A^nB^n}] = 0$ for the same reason. The operator $M_{A^nB^n}^{x,\delta }$ is permutation-invariant by construction. Then, we have

62 $$\begin{aligned} M^{x,\delta \ \dagger }_{A^nB^n} M^{x,\delta }_{A^nB^n}&= \sum _{\begin{array}{c} k,\ell ,\lambda ,\mu ,\\ k',\ell ',\lambda ',\mu '\;:\\ k-{\bar{H}}(\lambda )-\ell +{\bar{H}}(\mu ) \geqslant x - 4\delta \\ k'-{\bar{H}}(\lambda ')-\ell '+{\bar{H}}(\mu ') \geqslant x - 4\delta \end{array}} R^{k}_{A^nB^n}\,\varPi ^{\lambda }_{A^nB^n}\, \varPi ^{\mu }_{B^n}\, S^{\ell }_{B^n} S^{\ell '}_{B^n}\, \varPi ^{\mu '}_{B^n} \, \varPi ^{\lambda '}_{A^nB^n} \, R^{k'}_{A^nB^n} \nonumber \\&= \sum _{\begin{array}{c} k,k',\ell ,\lambda ,\mu \;:\\ k-{\bar{H}}(\lambda )-\ell +{\bar{H}}(\mu ) \geqslant x - 4\delta \\ k'-{\bar{H}}(\lambda )-\ell +{\bar{H}}(\mu ) \geqslant x - 4\delta \end{array}} R^{k}_{A^nB^n } \, \bigl (\varPi ^{\lambda }_{A^nB^n}\, \varPi ^{\mu }_{B^n}\, S^{\ell }_{B^n}\bigr ) \, R^{k'}_{A^nB^n} \nonumber \\&= \sum _{k,k'}\; R^k_{A^nB^n} \left( \sum _{\begin{array}{c} \ell ,\lambda ,\mu \,\\ k-{\bar{H}}(\lambda )-\ell +{\bar{H}}(\mu ) \geqslant x - 4\delta \\ k'-{\bar{H}}(\lambda )-\ell +{\bar{H}}(\mu ) \geqslant x - 4\delta \end{array}} \varPi ^{\lambda }_{A^nB^n}\, \varPi ^{\mu }_{B^n}\, S^{\ell }_{B^n} \right) R^{k'}_{A^nB^n} \nonumber \\&\leqslant \sum _{k,k'}\; R^k_{A^nB^n} R^{k'}_{A^nB^n} \nonumber \\&= \sum _{k}\; R^k_{A^nB^n} = \mathbb {1}_{A^nB^n} \end{aligned}$$

recalling that the operators $(\varPi ^\lambda _{A^nB^n}, \varPi ^\mu _{B^n}, S^\ell _{B^n})$ form a commuting set of projectors, and where in the third line the inner sum is taken to be the zero operator if no triplet $(\ell ,\lambda ,\mu )$ satisfies the given constraints. This shows Property (i).

Now, consider any state $|{\rho }\rangle _{ABR}$, where *R* is any reference system, and assume that ${D}(\rho _{AB}\,\Vert \,\Gamma _{AB}) - {D}(\rho _B\,\Vert \,\Gamma '_B) \geqslant x$. Rewrite this condition as

63 $$\begin{aligned} x \leqslant -H(\rho _{AB}) - {\text {tr}}[\rho _{AB}\ln \Gamma _{AB}] + H(\rho _B) + {\text {tr}}[\rho _B\ln \Gamma '_B]\ . \end{aligned}$$

We write64where we define 6566a further noting that the conditions in the sum defining $\blacksquare _1$ indeed imply that $k-{\bar{H}}(\lambda )-\ell +{\bar{H}}(\mu ) \geqslant -{\text {tr}}[\rho _{AB}\ln \Gamma _{AB}] - H(\rho _{AB}) + {\text {tr}}[\rho _{B}\ln \Gamma '_{B}] + H(\rho _{B}) - 4\delta \geqslant x - 4\delta$. We first consider $\blacksquare _1$. Define the projectors

66b $$\begin{aligned} X_1&= \sum _{k\geqslant -\!{\text {tr}}[\rho _{AB}\ln \Gamma _{AB}] - \delta } R^k_{A^nB^n}\ ;&X_1^\perp&= \mathbb {1}- X_1\ ; \end{aligned}$$

67a $$\begin{aligned} X_2&= \sum _{{\bar{H}}(\lambda ) \leqslant H(\rho _{AB}) + \delta } \varPi _{A^nB^n}^\lambda \ ;&X_2^\perp&= \mathbb {1}- X_2\ ; \end{aligned}$$

67b $$\begin{aligned} X_3&= \sum _{{\bar{H}}(\mu ) \geqslant H(\rho _{B}) - \delta } \varPi _{B^n}^\mu \ ;&X_3^\perp&= \mathbb {1}- X_3\ ; \end{aligned}$$

67c $$\begin{aligned} X_4&= \sum _{\ell \leqslant -\!{\text {tr}}[\rho _{B}\ln \Gamma '_{B}] + \delta } S^\ell _{B^n}\ ;&X_4^\perp&= \mathbb {1}- X_4\ , \end{aligned}$$

and observe that

67d $$\begin{aligned} {\text {Re}} \left\{ \ \blacksquare _1\ \right\} \ =\ {\text {Re}} \left\{ \langle {\rho }|_{ABR}^{\otimes n} \; \bigl (\;X_4\;X_3\;X_2\;X_1\;\bigr ) \; |{\rho }\rangle _{ABR}^{\otimes n} \right\} \ . \end{aligned}$$

Thanks to Proposition 2.2, we have $\Vert {\;X_1^\perp \; |{\rho }\rangle _{ABR}^{\otimes n}}\Vert \leqslant 2\exp (-n\eta /2)$, recalling that $\Vert {P|{\psi }\rangle }\Vert = \sqrt{{\text {tr}}[P\psi ]}$, and hence

68 $$\begin{aligned}&{\text {Re}} \left\{ \langle {\rho }|_{ABR}^{\otimes n} \; X_4\;X_3\;X_2\;X_1 \; |{\rho }\rangle _{ABR}^{\otimes n} \right\} \nonumber \\&\quad = {\text {Re}} \left\{ \langle {\rho }|_{ABR}^{\otimes n} \; X_4\;X_3\;X_2 \; |{\rho }\rangle _{ABR}^{\otimes n} \right\} - {\text {Re}} \left\{ \langle {\rho }|_{ABR}^{\otimes n} \; X_4\;X_3\;X_2\;X_1^\perp \; |{\rho }\rangle _{ABR}^{\otimes n} \right\} \nonumber \\&\quad \geqslant {\text {Re}} \left\{ \langle {\rho }|_{ABR}^{\otimes n} \; X_4\;X_3\;X_2 \; |{\rho }\rangle _{ABR}^{\otimes n} \right\} - 2\exp (-n\eta /2) \end{aligned}$$

using Cauchy–Schwarz to assert that ${\text {Re}}(\langle {\chi }\vert {\psi }\rangle ) \leqslant |{\langle {\chi }\vert {\psi }\rangle }|\leqslant \Vert {|{\chi }\rangle }\Vert \,\Vert {|{\psi }\rangle }\Vert$. Similarly, using Proposition 2.1, we have $\Vert {\;X_2^\perp \; |{\rho }\rangle _{ABR}^{\otimes n}}\Vert \leqslant {\text {poly}}(n)\exp (-n\eta /2)$. Also, we have $\Vert {\;X_3^\perp \; |{\rho }\rangle _{ABR}^{\otimes n}}\Vert \leqslant {\text {poly}}(n)\exp (-n\eta /2)$, and $\Vert {\;X_4^\perp \; |{\rho }\rangle _{ABR}^{\otimes n}}\Vert \leqslant 2\exp (-n\eta /2)$, yielding

69 $$\begin{aligned} {\text {Re}} \left\{ \langle {\rho }|_{ABR}^{\otimes n} \; X_4\;X_3\;X_2 \; |{\rho }\rangle _{ABR}^{\otimes n} \right\}&\geqslant {\text {Re}} \left\{ \langle {\rho }|_{ABR}^{\otimes n} \; X_4\;X_3 \; |{\rho }\rangle _{ABR}^{\otimes n} \right\} - {\text {poly}}(n)\,\exp (-n\eta /2)\ ; \end{aligned}$$

70 $$\begin{aligned} {\text {Re}} \left\{ \langle {\rho }|_{ABR}^{\otimes n} \; X_4\;X_3 \; |{\rho }\rangle _{ABR}^{\otimes n} \right\}&\geqslant {\text {Re}} \left\{ \langle {\rho }|_{ABR}^{\otimes n} \; X_4 \; |{\rho }\rangle _{ABR}^{\otimes n} \right\} - {\text {poly}}(n)\,\exp (-n\eta /2)\ ; \end{aligned}$$

71 $$\begin{aligned} {\text {Re}} \left\{ \langle {\rho }|_{ABR}^{\otimes n} \; X_4 \; |{\rho }\rangle _{ABR}^{\otimes n} \right\}&\geqslant 1 - 2\,\exp (-n\eta /2)\ . \end{aligned}$$

We take all these $\eta$’s to be the same, by choosing if necessary the minimum of the four possibly different $\eta$s. Hence, we have

72 $$\begin{aligned} {\text {Re}} \left\{ \ \blacksquare _1\ \right\} \ \geqslant \ 1 - {\text {poly}}(n)\,\exp (-n\eta /2)\ . \end{aligned}$$

Now we consider the term $\blacksquare _2$. We know that

73 $$\begin{aligned} \left\Vert { R^k_{A^nB^n} |{\rho }\rangle _{ABR}^{\otimes n} }\right\Vert&\leqslant \exp (-n\eta /2)&\text {if }k<-{\text {tr}}[\rho _{AB}\ln \Gamma _{AB}] - \delta \ ; \end{aligned}$$

74a $$\begin{aligned} \left\Vert { \varPi _{A^nB^n}^\lambda |{\rho }\rangle _{ABR}^{\otimes n} }\right\Vert&\leqslant {\text {poly}}(n)\exp (-n\eta /2)&\text {if }{\bar{H}}(\lambda ) > H(\rho _{AB}) + \delta \ ;\end{aligned}$$

74b $$\begin{aligned} \left\Vert { S^\ell _{B^n} |{\rho }\rangle _{ABR}^{\otimes n} }\right\Vert&\leqslant \exp (-n\eta /2)&\text {if }\ell >-{\text {tr}}[\rho _{B}\ln \Gamma '_{B}] + \delta \ ;\end{aligned}$$

74c $$\begin{aligned} \left\Vert { \varPi _{B^n}^\mu |{\rho }\rangle _{ABR}^{\otimes n} }\right\Vert&\leqslant {\text {poly}}(n)\exp (-n\eta /2)&\text {if }{\bar{H}}(\mu ) < H(\rho _{B}) - \delta \end{aligned}$$

recalling that $\Vert {P|{\psi }\rangle }\Vert = \sqrt{{\text {tr}}[P\psi ]}$. So, for each term in the sum (66b), we have

74d $$\begin{aligned} \left|{ \langle {\rho }|_{ABR}^{\otimes n} \bigl (S_{B^n}^\ell \varPi _{B^n}^\mu \varPi _{A^nB^n}^\lambda R_{A^nB^n}^k\bigr ) \, |{\rho }\rangle _{ABR}^{\otimes n} }\right|&= \left|{ \bigl (\langle {\rho }|_{ABR}^{\otimes n} S_{B^n}^\ell \varPi _{B^n}^\mu \varPi _{A^nB^n}^\lambda \bigr ) \bigl (R_{A^nB^n}^k \, |{\rho }\rangle _{ABR}^{\otimes n}\bigr ) }\right|\nonumber \\&\leqslant \left\Vert {\; R_{A^nB^n}^k \, |{\rho }\rangle _{ABR}^{\otimes n}\; }\right\Vert \cdot \left\Vert {\; \bigl (S_{B^n}^\ell \varPi _{B^n}^\mu \varPi _{A^nB^n}^\lambda \bigr ) \, |{\rho }\rangle _{ABR}^{\otimes n}\; }\right\Vert \nonumber \\&\leqslant {\text {poly}}(n)\,\exp (-n\eta /2) \end{aligned}$$

using the Cauchy–Schwarz inequality and because at least one of the four conditions is violated, causing at least one of the two the norms to decay exponentially (noting also that $S_{B^n}^\ell , \varPi _{B^n}^\mu , \varPi _{A^nB^n}^\lambda$ all commute). Because there are only at most ${\text {poly}}(n)$ terms, we have75Hence, we have

76 $$\begin{aligned} {\text {Re}} \left\{ \langle {\rho }|_{ABR}^{\otimes n} \, M^{x,\delta }_{A^nB^n} \, |{\rho }\rangle _{ABR}^{\otimes n} \right\} \&=\ {\text {Re}} \left\{ \ \blacksquare _1\ \right\} \ +\ {\text {Re}} \left\{ \ \blacksquare _2\ \right\} \nonumber \\&\geqslant \ {\text {Re}} \left\{ \ \blacksquare _1\ \right\} \ -\ \left|{\ \blacksquare _2\ }\right|\nonumber \\&\geqslant \ 1 - {\text {poly}}(n)\exp (-n\eta /2) \end{aligned}$$

proving Property (ii) for $\xi =\eta /2$. Note that $\xi$ does not depend on the state $|{\sigma }\rangle _{XR}$. Now, we prove Property (iii). Using Lemma B.1 and dropping some subsystem indices for readability, we have

77 $$\begin{aligned}&{\text {tr}}_{A^n} \bigl [ M^{x,\delta }_{A^nB^n} \Gamma _{AB}^{\otimes n} \bigl (M^{x,\delta }_{A^nB^n}\bigr )^\dagger \bigr ] \nonumber \\&\leqslant {\text {poly}}(n) \sum _{\begin{array}{c} k,\ell ,\lambda ,\mu \;:\\ k-{\bar{H}}(\lambda )-\ell +{\bar{H}}(\mu ) \geqslant x-4\delta \end{array}} {\text {tr}}_{A^n} \left[ S^\ell \varPi ^\mu \varPi ^\lambda R^k \, \Gamma ^{\otimes n} \, R^k \varPi ^\lambda \varPi ^\mu S^\ell \right] \ . \end{aligned}$$

Recall that, using Proposition 2.2 and Lemma 2.2,

78 $$\begin{aligned} R_{A^nB^n}^k \, \Gamma _{AB}^{\otimes n}&\leqslant {e}^{-nk}\, R_{A^nB^n}^k\leqslant {e}^{-nk}\, \mathbb {1}_{A^nB^n}\ ; \end{aligned}$$

79 $$\begin{aligned} \varPi ^\mu _{B^n} {\text {tr}}_{A^n} \left[ \varPi ^\lambda _{A^nB^n}\right] \varPi ^\mu _{B^n}&\leqslant {\text {poly}}(n)\,\exp (n({\bar{H}}(\lambda )-{\bar{H}}(\mu )))\,\mathbb {1}_{B^n}\ ; \end{aligned}$$

80 $$\begin{aligned} S_{B^n}^\ell&\leqslant {e}^{n\ell }\; S_{B^n}^\ell \, \Gamma _{B}'^{\otimes n} \leqslant {e}^{n\ell }\; \Gamma _{B}'^{\otimes n} \end{aligned}$$

further recalling that $[R_{A^nB^n}^k, \Gamma _{AB}^{\otimes n}] = 0$ and $[S_{B^n}^\ell , \Gamma _{B}'^{\otimes n}] = 0$. Combining these together yields

81 $$\begin{aligned} (78)&\leqslant {\text {poly}}(n) \sum _{\begin{array}{c} k,\ell ,\lambda ,\mu \;:\\ k-{\bar{H}}(\lambda )-\ell +{\bar{H}}(\mu ) \geqslant x-4\delta \end{array}} {e}^{-nk}\, S^\ell \, \varPi ^\mu \, {\text {tr}}_{A^n}\bigl [\varPi ^\lambda _{A^nB^n}\bigr ] \, \varPi ^\mu \, S^\ell \nonumber \\&\leqslant \sum _{\begin{array}{c} k,\ell ,\lambda ,\mu \;:\\ k-{\bar{H}}(\lambda )-\ell +{\bar{H}}(\mu ) \geqslant x-4\delta \end{array}} {\text {poly}}(n)\,{e}^{-nk+n({\bar{H}}(\lambda )-{\bar{H}}(\mu ))}\, S^\ell _{B^n} \nonumber \\&\leqslant \sum _{\begin{array}{c} k,\ell ,\lambda ,\mu \;:\\ k-{\bar{H}}(\lambda )-\ell +{\bar{H}}(\mu ) \geqslant x-4\delta \end{array}} {\text {poly}}(n)\,{e}^{-n(k-{\bar{H}}(\lambda )+{\bar{H}}(\mu )-\ell )}\, \Gamma _{B}'^{\otimes n} \nonumber \\&\leqslant {\text {poly}}(n)\,{e}^{-n(x-4\delta )}\, \Gamma _{B}'^{\otimes n}\ . \end{aligned}$$

Finally, suppose that $[\Gamma _{AB}, \Gamma _B'] = 0$, meaning that we can choose a simultaneous eigenbasis for $\Gamma _{AB}$ and $\Gamma _{B'}$. Then the operator $M_{A^nB^n}^{x,\delta }$ is a projector, as can be seen in (62) since in that case $\{ S_{B^n}^\ell \}, \{ \varPi ^\mu _{B^n} \}, \{ \varPi ^\lambda _{A^nB^n} \}, \{ R^k_{A^nB^n} \}$ are all complete sets of projectors all elements of which commute pairwise between different sets. Furthermore, $\Gamma _{B'}^{\otimes n}$ and $\Gamma _{AB}^{\otimes n}$ both commute with all of these projectors and therefore also with $M_{A^nB^n}^{x,\delta }$. $\square$

## Construction #3: Thermal Operations

### Statement and proof sketch

We now present a construction of a universal thermodynamic implementation of a time-covariant i.i.d. process, using the framework of thermal operations instead of Gibbs-preserving maps.

#### Theorem 7.1

Let *X* be a quantum system, $H_X$ a Hermitian operator, $\beta \geqslant 0$, ${\mathscr {E}}_{X\rightarrow X}$ a completely positive, trace-preserving map satisfying

82 $$\begin{aligned} {\mathscr {E}}_{X\rightarrow X}({e}^{-iH_Xt}\,(\cdot )\,{e}^{iH_Xt}) = {e}^{-iH_Xt}\,{\mathscr {E}}_{X\rightarrow X}(\cdot )\,{e}^{iH_Xt} \qquad \text {for all }t\in {\mathbb {R}}. \end{aligned}$$

Let $\epsilon >0$. Let $\delta >0$ be small enough and $n\in {\mathbb {N}}$ be large enough. Then, there exists an information battery *W*, a thermal operation $\varPhi _{X^nW}$, and battery states $\tau _W^{(\mathrm {i})}$ and $\tau _W^{(\mathrm {f})}$ such that:

(i) The effective work process ${\mathscr {T}}_{X^n\rightarrow X^n}$ associated with $\varPhi _{X^nW}$ and $\left( \tau _W^{(\mathrm {i})}, \tau _W^{(\mathrm {f})}\right)$ satisfies
  83 $$\begin{aligned} \frac{1}{2}\Vert { {\mathscr {T}}_{X^n\rightarrow X^n} - {\mathscr {E}}_{X\rightarrow X'}^{\otimes n} }\Vert _\diamond \leqslant \epsilon \ ; \end{aligned}$$
(ii) The work cost per copy satisfies
  84 $$\begin{aligned} \lim _{\delta \rightarrow 0} \lim _{n\rightarrow \infty } \frac{1}{n}\left[ w\left( \tau _W^{\mathrm {(i)}}\right) - w\left( \tau _W^{\mathrm {(f)}}\right) \right] = T({\mathscr {E}})\ . \end{aligned}$$

The main idea in the present construction is to first carry out a Stinespring dilation unitary explicitly using suitable ancillas as the environment system, and then to apply a conditional erasure process that resets the ancillas to a standard state while using the output of the process as side information. The idea of implementing a process in this fashion was also employed in Ref. [13].

**Fig. 1:**
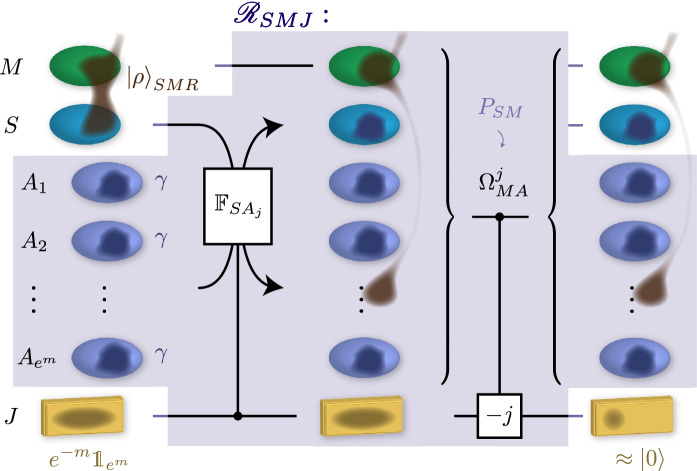
Construction of the thermal operation for universal conditional erasure using position-based decoding [19], illustrating the construction in the proof of Proposition 7.1 and Lemma 7.1. We define a map ${\mathscr {R}}_{SMJ}$ that acts on a system *S* to reset, a quantum memory *M* and a register *J*, which is promised to be initialized in the uniformly mixed state $e^{-m}\mathbb {1}_{e^{m}}$ of rank $e^m$ for a fixed and known value of *m*. A state $\rho _{SM}$ of the system and the memory is purified by a reference system *R* (not pictured). The map ${\mathscr {R}}_{SMJ}$ outputs the system *S* in a state close to the thermal state $\gamma _S$ and the register *J* in a state close to the pure state $|{0}\rangle _J$, all while ensuring that $\rho _{MR}$ remains unchanged (up to small errors), for all states $\rho _{SM}$ in a given class of states ${\mathscr {S}}_{SM}$. The routine is provided a POVM effect $P_{SM}$ whose task is to distinguish $\rho _{SM}$ from $\gamma _S\otimes \rho _M$ in a hypothesis test for all $\rho _{SM}\in {\mathscr {S}}_{SM}$. As long as *m* is not too large (as determined by how well $P_{SM}$ can perform this distinguishing), the procedure completes successfully. To implement ${\mathscr {R}}_{SMJ}$ (shaded region) we involve ${e}^{m}$ ancillas $A = A_1\ldots A_{{e}^{m}}$ with $A_j \simeq S$, each initialized in the thermal state $\gamma _{A_j} = \gamma _S$. Then *S* and $A_j$ are coherently swapped (${\mathbb {F}}_{SA_j}$) conditioned on the value stored in *J*. If *m* is not too large, a POVM $\{ \varOmega ^j_{MA} \}$ can infer the value *j* stored in *J*, up to a small error; the POVM is constructed from $P_{SM}$. We then coherently reset the *J* register to zero by conditioning on this outcome (up to a small error). The full procedure is a thermal operation where the ancillas are the heat bath and *J* is an information battery such that *m* work has been extracted in units of pure nats (see main text)

**Fig. 2:**
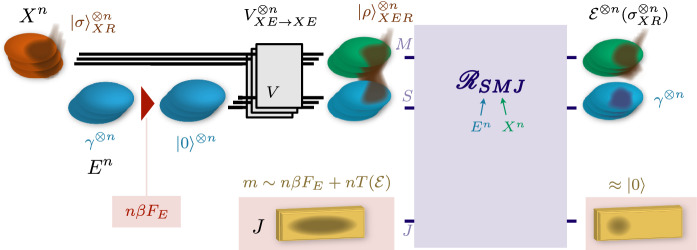
The conditional erasure procedure in Figure 1 can be used to construct an i.i.d. implementation of a given time-covariant process (Theorem 7.1). First we apply an energy-conserving Stinespring dilation of the process on all input copies, using a zero-initialized ancilla as environment system *E* for each copy. We then invoke the conditional erasure procedure ${\mathscr {R}}_{E^nX^nJ}$ to reset $E^n$ to the thermal state $\gamma _E^{\otimes n}$ using $X'^n$ as a memory, while extracting work using an information battery *J*. Here, the projector that can distinguish $\rho _{EX'}^{\otimes n}$ from $\mathbb {1}_{E^n}\otimes \rho _{X'^n}$ is the universal conditional typical projector given by Proposition E.2. The fact that ${\mathscr {R}}_{E^nX^nJ}$ preserves the correlations $[{\mathscr {E}}(\sigma _{XR})]^{\otimes n}$ between the memory (output systems $X'^n$) and the reference $R^n$ ensures that the process is implemented accurately. The amount of work extracted by ${\mathscr {R}}_{E^nX^nJ}$ is $m \sim n[\beta F_E+T({\mathscr {E}})]$ but $\sim n \beta F_E$ work has to be paid to prepare the initially pure $E^n$ ancillas, where $\beta F_E=-\ln {\text {tr}}({e}^{-\beta H_E})$. The overall work extracted is $\sim T({\mathscr {E}})$ per copy

Our core technical contribution for Construction #3 is to show how to build a thermodynamic protocol for universal conditional erasure, using the idea of position-based decoding [19, 49–55]. The assembly of the full thermal operation is slightly more involved than Constructions #1 and #2, because we cannot use Proposition 3.1. The construction will be illustrated in Figure 2, using a conditional erasure primitive whose construction is illustrated in Figure 1.

### Universal conditional erasure

Conditional erasure is a task that is of independent interest because it generalizes Landauer’s erasure principle to situations where a quantum memory is available. A protocol for thermodynamic conditional erasure of a system using a memory as quantum side information was given in ref. [56] for trivial Hamiltonians. Here, we study the problem of finding a universal protocol for conditional erasure, whose accuracy is guaranteed for any input state on *n* copies of a system, and where the system and memory Hamiltonians can be arbitrary.

#### Definition 7.1

*(Universal conditional erasure)*. Consider two systems *S*, *M*. Let $\sigma _S$ be a fixed state, let ${\mathscr {S}}_{SM} = \{ \rho _{SM} \}$ be an arbitrary set of states on $S\otimes M$, and let $\delta '\geqslant 0$. A *universal conditional* $\delta '$-*erasure process* of *S* using *M* as side information is a completely positive, trace non-increasing map ${\mathscr {T}}_{SM\rightarrow SM}$ such that for all $\rho _{SM}\in {\mathscr {S}}_{SM}$, and writing $|{\rho }\rangle _{SMR}$ a purification of $\rho _{SM}$, we have

85 $$\begin{aligned} F\bigl ( {\mathscr {T}}_{SM\rightarrow SM}(\rho _{SMR}), \sigma _S \otimes \rho _{MR} \bigr ) \geqslant 1 - \delta '\ . \end{aligned}$$

We provide a thermodynamic protocol for universal conditional erasure.

#### Proposition 7.1

Let *S*, *M* be systems with Hamiltonians $H_S,H_M$ and let $\gamma _S$ refer to the thermal state on *S*. Let ${\mathscr {S}}_{SM}$ be an arbitrary set of states on $S\otimes M$. Let $m\geqslant 0$ such that $e^m$ is integer. Let $P_{SM}$ be a Hermitian operator satisfying $0\leqslant P_{SM}\leqslant \mathbb {1}$ and $[P_{SM}, H_S+H_M]=0$, and assume that there exists $\kappa ,\kappa '\geqslant 0$ such that for all $\rho _{SM}\in {\mathscr {S}}_{SM}$ we have

86 $$\begin{aligned} {\text {tr}}\bigl [P_{SM}\,\rho _{SM}\bigr ]&\geqslant 1 - \kappa \ ; \end{aligned}$$

87a $$\begin{aligned} {\text {tr}} \left[ P_{SM}\, \left( \gamma _S\otimes \rho _{M}\right) \right]&\leqslant \frac{\kappa '}{e^m}\ . \end{aligned}$$

Then, there exists a thermal operation ${\mathscr {R}}_{SMJ\rightarrow SMJ}$ acting on the systems *SM* and an information battery *J*, such that the effective work process ${\mathscr {T}}_{SM\rightarrow SM}$ of ${\mathscr {R}}_{SMJ\rightarrow SMJ}$ with respect to the battery states $(\tau _J^{m},|{0}\rangle _J)$ is a universal conditional $(2\kappa +4\kappa ')$-erasure process with $\sigma _S=\gamma _S$ for the set of states ${\mathscr {S}}_{SM}'$, where ${\mathscr {S}}_{SM}'$ is the convex hull of ${\mathscr {S}}_{SM}$.

The proof of Proposition 7.1 is developed in the rest of this section. We start by reformulating the ideas of the convex-split lemma, the position-based decoding, and the catalytic decoupling schemes [19, 49–55] to form a protocol for universal conditional erasure. The underlying ideas of the following proposition are the same as, e.g., in Ref. [19]. Yet, our technical statement differs in some aspects and that is why we provide a proof for completeness. The setting is depicted in Fig. 1.

#### Lemma 7.1

(Conditional erasure unitary using position-based decoding). Consider two systems *S*, *M* and fix $m\geqslant 0$ such that $e^m$ is integer. Let *J* be a large register of dimension at least $2e^m$, and choose a fixed basis $\{|{j}\rangle _J\}$. Now, let $\gamma _S$ be any state, ${\mathscr {S}}_{SM}$ an arbitrary set of quantum states on $S\otimes M$, $P_{SM}$ a Hermitian operator satisfying $0\leqslant P_{SM} \leqslant \mathbb {1}$, and assume that there exists $\kappa ,\kappa '\geqslant 0$ such that for all $\rho _{SM}\in {\mathscr {S}}_{SM}$ the conditions (87) hold. Furthermore, let $A = A_1\otimes \cdots \otimes A_{e^m}$ be a collection of ancilla systems with each $A_j\simeq S$, and let $A' = A'_1\otimes \cdots \otimes A'_{e^m}$ be a copy of the full collection of ancilla systems. We write a purification of $\gamma _{A_j}$ on $A'_j$ as $|{\gamma }\rangle _{A_jA'_j} = \gamma _{A_j}^{1/2}\,|{\varPhi }\rangle _{A_j:A'_j}$. Let ${\mathscr {S}}_{SM}'$ be the convex hull of ${\mathscr {S}}_{SM}$. Then, there exists a unitary operator $W^{(m)}_{SMAJ\rightarrow SMAJ}$ satisfying the following property: For any reference system *R*, for any pure tripartite state $|{\rho }\rangle _{SMR}$ with $\rho _{SM}\in {\mathscr {S}}_{SM}'$, and for any $|{j}\rangle _J$ with $1\leqslant j\leqslant e^m$, we have

87b $$\begin{aligned} {\text {Re}} \left\{ \bigl ( \langle {{\hat{\tau ^j}}(\rho _{SMR})}|_{RMSAA'}\otimes \langle {0}|_J \bigr ) \; W^{(m)}_{SMAJ} \; \bigl (|{\rho }\rangle _{RMS}\otimes |{\gamma }\rangle _{A_\cdot A'_\cdot }^{\otimes e^m}\otimes |{j}\rangle _J\bigr ) \right\} \geqslant 1 - (2\kappa +4\kappa ')\ , \end{aligned}$$

where we have defined

88 $$\begin{aligned} |{{\hat{\tau ^j}}(\rho _{SMR})}\rangle _{RMSAA'} = |{\rho }\rangle _{A_jMR}\otimes |{\gamma }\rangle _{SA'_j}\otimes [|{\gamma }\rangle ^{\otimes ({e^m}-1)}]_{AA'\setminus A_jA'_j} \end{aligned}$$

and by the notation $AA'\setminus A_jA'_j$ we refer to all $AA'$ systems except $A_j A'_j$. Moreover, for any observables $H_S$, $H_M$ such that $[P_{SM}, H_S+H_M] = 0$, the unitary $W^{(m)}_{SMAJ}$ may be chosen such that $[H_S + H_M + \sum H_{A_j}, W^{(m)}_{SMAJ}] = 0$, where $H_{A_j} = H_S$.

Intuitively, we absorb the initial randomness present in the register *J*, e.g., given to us by the environment in a mixed state, and return it in a pure state; *J* can therefore be identified as an information battery. Similarly, *A* can be identified as a heat bath.

#### Proof

First observe that we can assume ${\mathscr {S}}_{SM}$ to be a convex set, because any convex combination of states in ${\mathscr {S}}_{SM}$ also satisfies the conditions (87). For the rest of the proof we assume without loss of generality that ${\mathscr {S}}_{SM}={\mathscr {S}}'_{SM}$.

The operator *W* is defined in two steps. The first operation simply consists on conditionally swapping *S* with $A_j$, depending on the value stored in *J*. Then, we infer again from *MA* which *j* we swapped *S* with, in order to coherently reset the register *J* back to the zero state (approximately). We define the first unitary operation as $W^{(1)}$, acting on systems *SAJ*

89 $$\begin{aligned} W^{(1)}_{SAJ} = \sum _{j} {\mathbb {F}}_{SA_{j}}\otimes |{j}\rangle \langle {j}|_J\ , \end{aligned}$$

where ${\mathbb {F}}_{SA_j}$ denotes the swap operator between the two designated systems. Observe that $W^{(1)}$ maps $\rho$ onto ${\hat{\tau ^j}}$ according to

90 $$\begin{aligned}&W^{(1)}_{SQJ} \left( |{\rho }\rangle _{RMS}\otimes |{\gamma }\rangle _{A_\cdot A'_\cdot }^{\otimes {e^m}}\otimes |{j}\rangle _J \right) \nonumber \\&\quad = |{\rho }\rangle _{RMA_j} \otimes |{\gamma }\rangle _{SA_j'} \otimes \left[ |{\gamma }\rangle ^{\otimes ({e^m}-1)}\right] _{AA'\setminus A_jA_j'} \otimes |{j}\rangle _J \nonumber \\&\quad = |{{\hat{\tau ^j}}}\rangle _{SRMAA'}\otimes |{j}\rangle _J\ . \end{aligned}$$

The second step is more tricky. We need to infer from the systems *MA* alone which *j* was stored in *J*. Fortunately the answer is provided in the form of position-based decoding [19], using a pretty good measurement. Define

91 $$\begin{aligned} \varLambda ^j_{MA} = P_{MA_j}\otimes \mathbb {1}_{A\setminus A_j} \end{aligned}$$

such that $\{\varLambda ^j_{MA}\}$ is a set of positive operators. We can form a POVM $\{ \varOmega ^j_{MA} \}_j \cup \{ \varOmega ^\perp _{MA} \}$ by normalizing the $\varLambda ^j$’s as follows:

92 $$\begin{aligned} \varOmega ^j_{MA}&= \varLambda _{MA}^{-1/2}\,\varLambda ^j_{MA}\,\varLambda _{MA}^{-1/2}\ ;&\varLambda _{MA}&= \sum _j \varLambda ^j_{MA}\ ;&\varOmega ^\perp _{MA}&= \mathbb {1}- \sum _j \varOmega ^j_{MA}\ . \end{aligned}$$

We would now like to lower bound ${\text {tr}}[\varOmega ^j_{MA}{\hat{\tau ^j}}_{MA}]$. Following the proof of [19, Theorem 2], we first invoke the Hayashi–Nagaoka inequality [57], which states that for any operators $0\leqslant A \leqslant \mathbb {1}$, $B\geqslant 0$, we have

93 $$\begin{aligned} \mathbb {1}- (A+B)^{-1/2}\,A\,(A+B)^{-1/2} \leqslant 2(\mathbb {1}- A) + 4B\ . \end{aligned}$$

Applying this inequality with $A = \varLambda ^j_{MA}$ and $B=\sum _{j'\ne j} \varLambda ^{j'}_{MA}$ we obtain

94 $$\begin{aligned} {\text {tr}} \left[ \left( \mathbb {1}-\varOmega ^j\right) {\hat{\tau ^j}}_{MA}\right]&\leqslant 2 {\text {tr}} \left[ \left( \mathbb {1}- \varLambda ^j_{MA}\right) {\hat{\tau ^j}}_{MA}\right] + 4\sum _{j'\ne j}{\text {tr}} \left[ \varLambda ^{j'}_{MA}{\hat{\tau ^j}}_{MA}\right] \nonumber \\&\leqslant 2 {\text {tr}} \left[ \left( \mathbb {1}- P_{SM}\right) \rho _{SM}\right] + 4m\,{\text {tr}} \left[ P_{SM} \left( \gamma _S\otimes \rho _M\right) \right] \nonumber \\&\leqslant 2\kappa + 4\kappa '\ . \end{aligned}$$

Now, let $\textsf {SHIFT}_J(x) = \sum _j |{j+x}\rangle \langle {j}|_J$ denote the SHIFT operation on the *J* register, modulo ${e^m}$; note that $\bigl ( \textsf {SHIFT}_J(x) \bigr )^\dagger = \textsf {SHIFT}_J(-x)$. We define

95 $$\begin{aligned} W^{(2)}_{MAJ}&= \left( \sum _{j} \varOmega ^{j}_{MA}\otimes \textsf {SHIFT}_J(-j) \right) \ ;&W'_{SMAJ}&= W^{(2)}_{MAJ} W^{(1)}_{SAJ} \end{aligned}$$

and we see that $W'^\dagger W'\leqslant \mathbb {1}$ thanks to Proposition B.3. Then, we have

96 $$\begin{aligned}&W'_{SMAJ} \, \left( |{\rho }\rangle _{RMS}\otimes |{\phi }\rangle _{A_\cdot A'_\cdot }^{\otimes {e^m}}\otimes |{j}\rangle _J\right) \nonumber \\&\quad = \left( \sum _{j'} \varOmega ^{j'}_{MA}\otimes \textsf {SHIFT}_J(-j') \right) \; \left( |{{\hat{\tau ^j}}}\rangle _{SRMAA'}\otimes |{j}\rangle _J \right) \nonumber \\&\quad = \sum _{j'} \left( \varOmega ^{j'}_{MA}\,|{{\hat{\tau ^j}}}\rangle _{RMSAA'} \right) \otimes |{j-j'}\rangle \ . \end{aligned}$$

Thanks to Proposition C.1, the operator $W'_{SMAJ}$ can be completed to a full unitary $W_{SMAJ}$ by using an extra qubit in the *J* register, and such that $\langle {0}|_J W_{SMAJ} |{j}\rangle _J = \langle {0}|_J W'_{SMAJ} |{j}\rangle _J$ for all $j=1,\ldots ,{e^m}$ (with the convention that $|{j}\rangle _J$ for $j\leqslant {e^m}$ forces the extra qubit to be in the zero state). So, recalling (95),

97 $$\begin{aligned}&\left( \langle {{\hat{\tau ^j}}}|_{RMSAA'}\otimes \langle {0}|_J \right) W_{SMAJ} \, \left( |{\rho }\rangle _{RMS}\otimes |{\phi }\rangle _{A_\cdot A'_\cdot }^{\otimes {e^m}}\otimes |{j}\rangle _J\right) \nonumber \\&\quad = \left( \langle {{\hat{\tau ^j}}}|_{RMSAA'}\otimes \langle {0}|_J \right) W'_{SMAJ} \, \left( |{\rho }\rangle _{RMS}\otimes |{\phi }\rangle _{A_\cdot A'_\cdot }^{\otimes {e^m}}\otimes |{j}\rangle _J\right) \nonumber \\&\quad = \langle {{\hat{\tau ^j}}}\vert {\varOmega ^j_{MA}}\vert {{\hat{\tau ^j}}}\rangle _{RMSAA'} \nonumber \\&\quad \geqslant 1 - (2\kappa +4\kappa ')\ . \end{aligned}$$

To prove the last part of the claim, let $H_S, H_M$ be observables such that $[P_{SM}, H_S+H_M] = 0$ and $[H_S, \gamma _S]=0$. Let $H_{A_j} = H_S$ and we write $H_A = \sum _j H_{A_j}$. For all *j*, we have

98 $$\begin{aligned} {[}H_S + H_M + H_A, \varLambda ^j_{MA}] = \bigl [H_S + {\textstyle \sum _{j'\ne j}} H_{A_{j'}}, \varLambda ^j_{MA}\bigr ] + [H_M + H_{A_j}, P_{MA_j}] = 0\ . \end{aligned}$$

This implies that $[H_S + H_M + H_A, \varLambda _{MA}] = 0$, and in turn $\bigl [H_S + H_M + H_A, \varLambda _{MA}^{-1/2}\bigr ] = 0$, and thus also $[H_S + H_M + H_A, \varOmega ^j] = 0$. Hence, we have

99 $$\begin{aligned} \bigl [H_S + H_M + H_A, W^{(2)}_{MAJ}\bigr ] = 0\ . \end{aligned}$$

Clearly, $[H_S + H_M + H_A, W^{(1)}_{SAJ}] = 0$, and hence $[H_S+H_M+H_A, W'_{SMAJ}] = 0$. Using Proposition C.2 instead of Proposition C.1, we may further enforce $[H_S + H_M + H_A, W_{SMAJ}] = 0$, as required. $\square$

We now give the proof of Proposition 7.1.

#### Proof

(Proposition 7.1). Let $W^{(m)}_{SMAJ}$ be the energy-conserving unitary as in Lemma 7.1 and define the thermal operation

100 $$\begin{aligned} {\mathscr {R}}_{SMJ}(\cdot ) = {\text {tr}}_{A}\bigl [ W^{(m)}_{SMAJ} \bigl ((\cdot )\otimes \gamma _{A}\bigr ) W^{(m)\,\dagger }_{SMAJ}\bigr ]\ . \end{aligned}$$

Identifying *J* as an information battery, the associated effective work process of ${\mathscr {R}}_{SMJ}$ with respect to $(\tau _J^m,|{0}\rangle _J)$ is

101 $$\begin{aligned} {\mathscr {T}}_{SM\rightarrow SM}(\cdot )&= {\text {tr}}_{A}\bigl [ \langle {0}|_J W^{(m)}_{SMAJ} \bigl ((\cdot )\otimes \gamma _A\otimes \tau _J^{m}\bigr ) W^{(m)\,\dagger }_{SMAJ} |{0}\rangle _J \bigr ]\ . \end{aligned}$$

Let $\rho _{SM}\in {\mathscr {S}}_{SM}'$ and let $|{\rho }\rangle _{SMR}$ be a purification of $\rho _{SM}$. We have that the state vector

102 $$\begin{aligned} e^{-m/2}\sum _{j=1}^{e^m} \langle {0}|_J W_{SMAJ}^{m}\bigl (|{\rho }\rangle _{SMR}\otimes |{\gamma }\rangle _{AA'}^{\otimes e^m}\otimes |{j}\rangle _J\bigr ) \otimes |{j}\rangle _{R_J} \end{aligned}$$

is a purification of ${\mathscr {T}}_{SM\rightarrow SM}(\rho _{SMR})$, where $R_J$ is an additional register. Similarly, the state vector

103 $$\begin{aligned} e^{-m/2}\sum _{j=1}^{e^m}|{{\hat{\tau ^j}}(\rho _{SMR})}\rangle _{RMSAA'}\otimes |{j}\rangle _{R_J} \end{aligned}$$

is a purification of $\gamma _S\otimes \rho _{MR}$. Then, with Uhlmann’s theorem we find

104 $$\begin{aligned}&F\bigl ( {\mathscr {T}}_{SM\rightarrow SM}(\rho _{SMR}) , \gamma _S\otimes \rho _{MR}\bigr ) \nonumber \\&\quad \geqslant e^{-m}\sum _{j=1}^{e^m} {\text {Re}} \left\{ \bigl ( \langle {{\hat{\tau ^j}}(\rho _{SMR})}|_{RMSAA'}\otimes \langle {0}|_J \bigr ) \; W^{(m)}_{SMAJ} \; \bigl (|{\rho }\rangle _{RMS}\otimes |{\gamma }\rangle _{A_\cdot A'_\cdot }^{\otimes e^m}\otimes |{j}\rangle _J\bigr ) \right\} \nonumber \\&\quad \geqslant 1 - (2\kappa +4\kappa ')\ , \end{aligned}$$

making use of (88). $\square$

### Construction via universal conditional erasure

This section is devoted to the proof of Theorem 7.1. The strategy is to exploit the fact that time-covariant processes admit a Stinespring dilation with an energy-conserving unitary using an environment system with a separate Hamiltonian. This property enables us to map the problem of implementing such a process directly to a conditional erasure problem with a system and memory that are non-interacting.

The following lemma formalizes the property of time-covariant processes we make use of. Various proofs of this lemma can be found in [58, 59, Appendix B] and [60, Theorem 25].

#### Lemma 7.2

(Stinespring dilation of covariant processes  [58–60]). Let *X* be a quantum system with Hamiltonian $H_X$, and ${\mathscr {E}}_{X\rightarrow X}$ be a completely positive, trace-preserving map that is covariant with respect to time evolution. That is, for all *t* we have

105 $$\begin{aligned} {\mathscr {E}}_{X\rightarrow X}({e}^{-iH_X t}\,(\cdot )\,{e}^{iH_X t}) = {e}^{-iH_X t}\, {\mathscr {E}}_{X\rightarrow X}(\cdot )\,{e}^{iH_X t} \ . \end{aligned}$$

Then, there exists a system *E* with Hamiltonian $H_E$ including an eigenstate $|{0}\rangle _E$ of zero energy, as well as a unitary $V_{EX\rightarrow EX}$ such that

106 $$\begin{aligned} {\mathscr {E}}_{X\rightarrow X}(\cdot ) = {\text {tr}}_E \left[ V\, \left( |{0}\rangle \langle {0}|_E \otimes (\cdot )\right) \,V^\dagger \right] \end{aligned}$$

as well as $V\, (H_X+H_E) \, V^\dagger = H_X + H_E$.

We provide an additional proof in “Appendix A”. The main idea behind the construction in the following proof of Theorem 7.1 is depicted in Fig. 2.

#### Proof

(Theorem 7.1) Thanks to Lemma 7.2, there exists an environment system *E* with Hamiltonian $H_E$, as well as an energy-conserving unitary $V_{XE}$ and a state $|{0}\rangle _E$ of zero energy such that (107) holds. Let $F_E = -\beta ^{-1}\ln (Z_E)$ with $Z_E = {\text {tr}}[{e}^{-\beta H_E}]$. We define

107 $$\begin{aligned} x = \min _\sigma \left\{ {D}(\sigma \,\Vert \,{e}^{-\beta H_X}) - {D}({\mathscr {E}}(\sigma )\,\Vert \,{e}^{-\beta H_X}) \right\} = - T({\mathscr {E}}) \ . \end{aligned}$$

Writing $\rho _{XE} = V_{XE} \left( |{0}\rangle \langle {0}|_E\otimes \sigma _X\right) V_{XE}^\dagger$, we have that $x = \min _{\sigma _X} \bigl \{ -{H}(\sigma _X) + \beta {\text {tr}}[\sigma _X H_X] + {H}(\rho _{X}) - \beta {\text {tr}}[\rho _X H_X] \bigr \}$. By ${\text {tr}}[\sigma _X H_X] = {\text {tr}}\bigl [(|{0}\rangle \langle {0}|_E\otimes \sigma _X)(H_X + H_E)\bigr ] = {\text {tr}}\bigl [\rho _{XE} \left( H_X+H_E\right) \bigr ]$, we see that

108 $$\begin{aligned} x = \min _{\sigma _X} \left\{ -{H}(\rho _{XE}) + {H}(\rho _{X}) + \beta {\text {tr}}[\rho _E H_E] \right\} \ . \end{aligned}$$

Observe that for any such $\rho _{XE}$, we have

109 $$\begin{aligned} -{H}(E\,|\,X)_{\rho } + \beta {\text {tr}}[\rho _E H_E]&\geqslant -{H}(E)_{\rho } + \beta {\text {tr}}[\rho _E H_E] + \ln (Z) - \ln (Z) \nonumber \\&= {D}(\rho _E\,\Vert \,\gamma _E) + \beta F_E \geqslant \beta F_E \end{aligned}$$

using the sub-additivity of the von Neumann entropy and the fact that relative entropy is positive for normalized states. Hence, we have $x\geqslant \beta F_E$.

Let

110 $$\begin{aligned} {\mathscr {S}}_{E^{n}X^{n}} = \bigl \{ \rho _{EX}^{\otimes n}\ :\ \rho _{EX} = V_{XE}(|{0}\rangle \langle {0}|_E\otimes \sigma _X)V_{XE}^\dagger \ \text {for some } \sigma _X \bigr \}\ , \end{aligned}$$

noting that for all $\rho _{EX}^{\otimes n}\in {\mathscr {S}}_{E^nX^n}$, we have ${D}(\rho _{EX}\,\Vert \,{e}^{-\beta (H_X+H_E)}) - {D}(\rho _X\,\Vert \,{e}^{-\beta H_X}) = {D}(\sigma \,\Vert \,{e}^{-\beta H_X}) - {D}({\mathscr {E}}(\sigma )\,\Vert \,{e}^{-\beta H_X}) \geqslant x$. Let $P_{E^nX^n}^{x,\delta }$ be the universal typical and relative conditional operator furnished by Proposition 6.1, where $\Gamma _X = {e}^{-\beta H_X}$ and $\Gamma _{XE} = {e}^{-\beta (H_X+H_E)} = \Gamma _X\otimes \Gamma _E$ with $\Gamma _E = {e}^{-\beta H_E}$. Since $\Gamma _{XE}$ commutes with $\mathbb {1}_E\otimes \Gamma _X$, Proposition 6.1 guarantees that $P_{E^nX^n}^{x,\delta }$ is a projector which furthermore commutes with $\Gamma _{XE}^{\otimes n}$ and $\Gamma _X^{\otimes n}$. We proceed to show that $P_{E^nX^n}^{x,\delta }$ can perform a hypothesis test between $\rho _{EX}^{\otimes n}$ and $\gamma _E^{\otimes n}\otimes \rho _X^{\otimes n}$. Recalling Definition 6.1 we have

111 $$\begin{aligned} {\text {tr}}\bigl [ P_{E^nX^n}^{x,\delta } \rho _{EX}^{\otimes n} \bigr ]&\geqslant 1- \kappa \ , \end{aligned}$$

with $\kappa = {\text {poly}}(n)\,{e}^{-n\eta }$ for some $\eta >0$ independent of $\rho$ and *n*. By construction we have $\mathbb {1}_X\otimes \Gamma _E = \Gamma _X^{-1/2}\Gamma _{XE}\Gamma _X^{-1/2}$, and so thanks to Point (iii) of Definition 6.1 we can compute

112 $$\begin{aligned} {\text {tr}}_{E^n}\bigl [ P_{E^nX^n}^{x,\delta } \Gamma _E^{\otimes n} \bigr ]&= \bigl (\Gamma _{X}^{-1/2}\bigr )^{\otimes n} {\text {tr}}_{E^n}\bigl [ P_{E^nX^n}^{x,\delta } \Gamma _{XE}^{\otimes n} \bigr ] \bigl (\Gamma _{X}^{-1/2}\bigr )^{\otimes n} \nonumber \\&\leqslant {\text {poly}}(n) \exp (-n(x-4\delta ))\,\mathbb {1}_{X^n}\ , \end{aligned}$$

where we furthermore used the fact that $P_{E^nX^n}^{x,\delta }$ commutes with $\Gamma _{XE}^{\otimes n}$ and with $\Gamma _{X}^{\otimes n}$. We therefore see using $\gamma _E = \Gamma _E/{\text {tr}}[\Gamma _E]$ that

113 $$\begin{aligned} {\text {tr}}\bigl [ P_{E^nX^n}^{x,\delta } \rho _X^{\otimes n}\otimes \gamma _E^{\otimes n} \bigr ]&\leqslant \frac{1}{{\text {tr}}[\Gamma _E^{\otimes n}]} \, {\text {poly}}(n)\exp \bigl (-n(x-4\delta )\bigr )\, {\text {tr}}\bigl [\rho _X^{\otimes n}\bigr ] \nonumber \\&= {\text {poly}}(n)\,\exp \bigl (-n(x - \beta F_E - 4\delta )\bigr )\ . \end{aligned}$$

Let

114 $$\begin{aligned} {e}^{m} = \bigl \lfloor \exp \bigl \{n(x - \beta F_E - 4\delta - \eta )\bigr \} \bigr \rfloor \ , \end{aligned}$$

such that ${\text {tr}}\bigl [ P_{E^nX^n}^{x,\delta } \rho _X^{\otimes n}\otimes \gamma _E^{\otimes n} \bigr ] \leqslant e^{-m}\kappa '$ by choosing $\kappa ' = {\text {poly}}(n){e}^{-n\eta }$.

Now let *J* be a register of dimension at least $2{e}^{m}$ and let ${\mathscr {R}}_{E^nX^nJ}$ be the thermal operation furnished by Proposition 7.1 for $S=E^n$, $M=X^n$, ${\mathscr {S}}_{E^nX^n}$, $P_{E^nX^n}^{x,\delta }$, *m*, $\kappa$, and $\kappa '$ as defined above. Here, we have assumed that $x > \beta F_E$, and that furthermore $\delta ,\eta$ are small enough such that $4\delta +\eta < (x-\beta F_E)$; if instead $x=\beta F_E$ then we can set $e^m=1$ and ${\mathscr {R}}_{E^nX^nJ}(\cdot ) = {\text {tr}}_{E^n}(\cdot )\otimes \gamma _E^{\otimes n}$ (which is a thermal operation) in the following.

We proceed to show that the effective work process ${\mathscr {T}}^{{\mathscr {R}}}_{E^nX^n\rightarrow E^nX^n}$ of ${\mathscr {R}}_{E^nX^nJ}$ with respect to $(\tau _J^m,|{0}\rangle _J)$ is close to the partial trace map ${\mathscr {T}}_{E^nX^n\rightarrow E^nX^n}^{(0)}(\cdot ) = {\text {tr}}_{E^n}(\cdot )\otimes \gamma _E^{\otimes n}$ in diamond distance. We invoke the post-selection technique (Theorem 2.1) to show this. Let $\zeta _{E^nX^n}$ be the de Finetti state which via (21) can be written as the convex combination of a finite number of i.i.d. states

115 $$\begin{aligned} \zeta _{E^nX^n} = \sum p_i \phi _i^{\otimes n}\ . \end{aligned}$$

Hence $\zeta _{E^nX^n}$ lies in the convex hull of ${\mathscr {S}}_{E^nX^n}$, and from Proposition 7.1 and Definition 7.1 we see that for a purification $|{\zeta }\rangle _{E^nX^nR}$ of $\zeta _{E^nX^n}$ we have

116 $$\begin{aligned} F\bigl ( {\mathscr {T}}^{{\mathscr {R}}}_{E^nX^n\rightarrow E^nX^n}(\zeta _{E^nX^nR}), \gamma _E^{\otimes n}\otimes {\text {tr}}_{E^n}(\zeta _{E^nX^nR}) \bigr ) \ge 1 - (2\kappa +4\kappa ')\ . \end{aligned}$$

Using $D(\rho ,\sigma ) \le \sqrt{1 - F(\rho ,\sigma )}$ along with Theorem 2.1 we find

117 $$\begin{aligned} \frac{1}{2}\Vert { {\mathscr {T}}^{{\mathscr {R}}}_{E^nX^n\rightarrow E^nX^n} - {\mathscr {T}}_{E^nX^n\rightarrow E^nX^n}^{(0)} }\Vert _{\diamond } \leqslant \sqrt{2\kappa + 4\kappa '} = {\text {poly}}(n)\,{e}^{-n\eta /2}\ . \end{aligned}$$

We can start piecing together the full process. Our overall protocol needs to (a) bring in a heat bath $E^n$, i.e., ancillas initialized in their thermal state, (b) prepare the states $|{0}\rangle _E^{\otimes n}$ on the ancillas using an auxiliary information battery (denoted by $W'$ below), (c) apply the energy-conserving unitary $V_{XE}^{\otimes n}$, (d) apply ${\mathscr {R}}_{E^nX^nJ}$ using an information battery *J* initialized in the state $\tau _J^m$, and (e) discard the ancillas.

As explained in Sect. 3, there exists a thermal operation ${{\widetilde{\varPhi }}}_{E^nW'}$ on the ancillas and an information battery $W'$ along with battery states $(\tau _{W'}^{(1)}, \tau _{W'}^{(2)})$ such that ${{\widetilde{\varPhi }}}_{E^nW'}( \gamma _E^{\otimes n} \otimes \tau _{W'}^{(1)} ) = |{0}\rangle \langle {0}|_E^{\otimes n}\otimes \tau _{W'}^{(2)}$ and with $w(\tau _{W'}^{(1)}) - w(\tau _{W'}^{(2)})$ arbitrarily close to $-\beta n F_E$. Now let $W = J\otimes W'$, $\tau _W^{(\mathrm {i})} = \tau _{W'}^{(1)}\otimes \tau _J^{m}$, $\tau _W^{(\mathrm {f})} = \tau _{W'}^{(2)}\otimes |{0}\rangle \langle {0}|_J$, and define

118 $$\begin{aligned} \varPhi _{X^nW}(\cdot )&= {\text {tr}}_{E^n}\Bigl [ {\mathscr {R}}_{E^nX^nJ}\Bigl ( V_{XE}^{\otimes n} \; {{\widetilde{\varPhi }}}_{E^nW'}\bigl ( (\cdot )\otimes \gamma _E^{\otimes n}\bigr ) \; (V_{XE}^{\otimes n})^\dagger \Bigr ) \Bigr ]\ . \end{aligned}$$

The map $\varPhi _{X^nW}$ is a thermal operation because it is a concatenation of thermal operations. The overall heat bath is formed of the systems $E^n$, the ancillas $A^n$ used in the implementation of ${\mathscr {R}}_{E^nX^nJ}$, as well as the implicit heat bath used in the implementation of ${\widetilde{\varPhi }}_{E^nW'}$. The system $W=J\otimes W'$ is the information battery. We can verify that the associated effective work process with respect to $\bigl (\tau _W^{(\mathrm {i})},\tau _W^{(\mathrm {f})}\bigr )$ is

119 $$\begin{aligned} {\mathscr {T}}_{X^n}(\cdot )&= \bigl \langle {0}\bigr |_J {\text {tr}}_{E^n}\Bigl [ {\mathscr {R}}_{E^nX^nJ}\Bigl ( V_{XE}^{\otimes n} \; {\text {tr}}_{W'}\bigl [P_{W'}^{(2)}\; {{\widetilde{\varPhi }}}_{E^nW'}\bigl ( (\cdot )\otimes \tau _{W'}^{(1)}\otimes \tau _J^{m}\otimes \gamma _E^{\otimes n} \bigr ) \bigr ] \; (V_{XE}^{\otimes n})^\dagger \Bigr ) \Bigr ] \bigl |{0}\bigr \rangle _J \nonumber \\&= {\text {tr}}_{E^n}\Bigl [ \bigl \langle {0}\bigr |_J {\mathscr {R}}_{E^nX^nJ}\Bigl ( \bigl [ V_{XE}^{\otimes n} \; \bigl ( (\cdot )\otimes |{0}\rangle \langle {0}|_E^{\otimes n} \bigr )\; (V_{XE}^{\otimes n})^\dagger \bigr ] \otimes \tau _J^{m} \Bigr ) \bigl |{0}\bigr \rangle _J \Bigr ] \nonumber \\&= {\text {tr}}_{E^n}\Bigl [ {\mathscr {T}}^{{\mathscr {R}}}_{E^nX^n}\Bigl ( V_{XE}^{\otimes n} \; \bigl ( (\cdot )\otimes |{0}\rangle \langle {0}|_E^{\otimes n} \bigr )\; (V_{XE}^{\otimes n})^\dagger \Bigr ) \Bigr ] \nonumber \\&= {\text {tr}}_{E^n}\Bigl [ V_{XE}^{\otimes n} \; \bigl ( (\cdot )\otimes |{0}\rangle \langle {0}|_E^{\otimes n} \bigr )\; (V_{XE}^{\otimes n})^\dagger \Bigr ] + \varDelta _{X^n}(\cdot ) \nonumber \\&= {\mathscr {E}}_{X\rightarrow X}^{\otimes n}(\cdot ) + \varDelta _{X^n}(\cdot )\ , \end{aligned}$$

where $\varDelta _{X^n}(\cdot ) = {\text {tr}}_{E^n}\bigl ( {\mathscr {T}}^{{\mathscr {R}}}_{X^nE^n}(\cdot ) - {\mathscr {T}}^{(0)}_{X^nE^n}(\cdot ) \bigr )$ satisfies $(1/2)\Vert {\varDelta _{X^n}}\Vert _\diamond \leqslant {\text {poly}}(n){e}^{-n\eta /2}$. Therefore for any fixed $\epsilon$ and for *n* large enough we have $(1/2)\Vert {{\mathscr {T}}_{X^n} - {\mathscr {E}}_{X\rightarrow X}^{\otimes n}}\Vert _\diamond \leqslant \epsilon$.

The associated work cost per copy satisfies

120 $$\begin{aligned} \lim _{\delta \rightarrow 0} \lim _{n\rightarrow \infty } \frac{1}{n}\bigl [w(\tau _W^{(\mathrm {i})}) - w(\tau _W^{(\mathrm {f})})\bigr ]&= \lim _{\delta \rightarrow 0} \lim _{n\rightarrow \infty } \frac{1}{n}\bigl [ w(\tau _{W'}^{(1)}) - w(\tau _{W'}^{(2)}) - m \bigr ] \nonumber \\&= \lim _{\delta \rightarrow 0} \lim _{n\rightarrow \infty } \frac{1}{n}\bigl [ -n\beta F_E - n(x-\beta F_E - 4\delta + \eta ) + \upsilon \bigr ] \nonumber \\&= T({\mathscr {E}})\ , \end{aligned}$$

recalling (115), where $0 \leqslant \upsilon \leqslant 2$ accounts for the rounding error in (115) and a possible arbitrarily small difference between $-n\beta F_E$ and $w(\tau _{W'}^{(1)}) - w(\tau _{W'}^{(2)})$, and recalling that $\eta \rightarrow 0$ as $\delta \rightarrow 0$. $\square$

## Discussion

Our results fits in the line of research extending results in thermodynamics from state-to-state transformations to quantum processes. Implementations of quantum processes are difficult to construct because they need to reproduce the correct correlations between the output and the reference system, and not only produce the correct output state. Here, we have seen that it is nevertheless possible to implement any quantum process at an optimal work cost: Any implementation that would use less work would violate the second law of thermodynamics on a macroscopic scale. As a special case this also provides an operational interpretation of the minimal entropy gain of a channel [35–42].

Our three constructions of optimal implementations of processes are valid in different settings, and it remains unclear if they can be unified in a single protocol that presents the advantages of all three constructions. Namely, is it possible to use a physically well-justified framework, e.g. thermal operations, to universally implement any i.i.d. process? We expect this to be possible only if an arbitrary amount of coherence is allowed, in analogy with the entanglement embezzling state required in the reverse Shannon theorem [22, 23].

Finally, the notion of quantum typicality that we have introduced in Definition 6.1 and Proposition 6.1 might be interesting in its own right. We anticipate that similar considerations might provide pathways to smooth other information-theoretic quantities [54, 61, 62] and to study the joint typicality conjecture [26, 63–66].

## A Missing proofs

### Proof

(Lemma 2.2). A useful expression for $\varPi ^\lambda _{A^nB^n}$ may be obtained following [25, Section V]

121 $$\begin{aligned} \varPi ^{\lambda }_{A^nB^n}&= \frac{\dim ({\mathscr {Q}}_\lambda )}{s_\lambda ({\text {diag}}(\lambda /n))} \int dU_{AB}\; \varPi ^\lambda _{A^nB^n} \left( U_{AB} \, {\text {diag}}(\lambda /n)_{AB} \, U_{AB}^{\dagger } \right) ^{\otimes n} \, \varPi ^\lambda _{A^nB^n} \nonumber \\&\leqslant {\text {poly}}(n)\,{e}^{n{\bar{H}}(\lambda )} \, \int dU_{AB}\; \left( U_{AB} \, {\text {diag}}(\lambda /n)_{AB} \, U_{AB}^{\dagger } \right) ^{\otimes n}\ , \end{aligned}$$

recalling that $\varPi ^\lambda _{A^nB^n}$ commutes with any i.i.d. state, with $s_\lambda (X) = {\text {tr}}[q_\lambda (X)]$ and using bounds on $\dim ({\mathscr {Q}}_\lambda )$ and $s_\lambda ({\text {diag}}(\lambda /n))$ derived in Ref. [25]. Here, $dU_{AB}$ denotes the Haar measure over all unitaries acting on ${\mathscr {H}}_{AB}$, normalized such that $\int dU_{AB} = 1$. We then have

122 $$\begin{aligned} {\text {tr}}_{A^n} \left[ \varPi ^\lambda _{A^nB^n}\right] \leqslant {\text {poly}}(n)\,{e}^{n{\bar{H}}(\lambda )} \int dU_{AB}\, {\text {tr}}_{A^n} \left[ \left( U_{AB}\,{\text {diag}}(\lambda /n)_{AB}\,U_{AB}^\dagger \right) ^{\otimes n} \right] \ . \end{aligned}$$

Observe that for any state $\omega _B$, we have

123 $$\begin{aligned} \bigl \Vert {\varPi ^{\lambda '}_{B^n}\,\omega _B^{\otimes n}\,\varPi ^{\lambda '}_{B^n}}\bigr \Vert _\infty&= \bigl \Vert { \;[\;{q_{\lambda '}(\omega _B)\otimes \mathbb {1}_{{\mathscr {P}}_{\lambda '}}}\;]_{\lambda '}\; }\bigr \Vert _\infty \nonumber \\&= \Vert { q_{\lambda '}(\omega _B) }\Vert _\infty \leqslant {\text {tr}} \left[ q_{\lambda '}(\omega _B)\right] \nonumber \\&\leqslant {\text {poly}}(n)\,{e}^{-n{\bar{H}}(\lambda ')} \end{aligned}$$

as derived e.g. in [25, Eq. (9)], and thus for any state $\omega _B$,

124 $$\begin{aligned} \varPi ^{\lambda '}_{B^n}\,\omega _B^{\otimes n}\,\varPi ^{\lambda '}_{B^n} \leqslant {\text {poly}}(n)\,{e}^{-n{\bar{H}}(\lambda ')}\, \varPi ^{\lambda '}_{B^n} \ . \end{aligned}$$

Hence, we get

125 $$\begin{aligned}&\varPi ^{\lambda '}_{B^n}\,{\text {tr}}_{A^n} \left[ \varPi ^\lambda _{A^nB^n}\right] \,\varPi ^{\lambda '}_{B^n} \nonumber \\&\quad \leqslant {\text {poly}}(n)\,{e}^{n{\bar{H}}(\lambda )} \int dU_{AB}\; \varPi ^{\lambda '}_{B^n} \, \left( {\text {tr}}_A \left[ U_{AB}\,{\text {diag}}(\lambda /n)_{AB}\,U_{AB}^\dagger \right] \right) ^{\otimes n} \varPi ^{\lambda '}_{B^n} \nonumber \\&\quad \leqslant {\text {poly}}(n)\,{e}^{n{\bar{H}}(\lambda )} \int dU_{AB}\; {\text {poly}}(n)\,{e}^{-n{\bar{H}}(\lambda ')}\, \varPi _{B^n}^{\lambda '} \nonumber \\&\quad = {\text {poly}}(n)\,{e}^{n({\bar{H}}(\lambda ) - {\bar{H}}(\lambda '))}\, \varPi _{B^n}^{\lambda '}\ , \end{aligned}$$

as required. $$\square $$

### Proof

(Proposition 2.1) The Fannes–Audenaert continuity bound [67, 68] of the entropy states that for any $\delta '>0$ there exists $\xi (\delta ')>0$ such that for any quantum states $\rho ,\sigma$ with $D(\rho ,\sigma )\leqslant \delta '$ we have

126 $$\begin{aligned} |{H(\rho ) - H(\sigma )}|\leqslant \xi (\delta ')\ , \end{aligned}$$

and furthermore $\xi (\delta ')$ is monotonically strictly decreasing and $\xi (\delta ')\rightarrow 0$ if $\delta '\rightarrow 0$. Now, let $\delta >0$, let $\xi ^{-1}$ be the inverse function of $\xi$, and let $\delta '=\xi ^{-1}(\delta )$. Consider the set of Young diagrams $\varLambda _{\delta '} = \{ \lambda \in {\text {Young}}(d_A,n) : D({\text {diag}}(\lambda /n),\rho ) \leqslant \delta ' \}$. For all $\lambda \in \varLambda _{\delta '}$, we have that $|{H(\rho ) - {\bar{H}}(\lambda )}|\leqslant \delta$ thanks to the Fannes–Audenaert inequality. Then, we have

127 $$\begin{aligned} {\text {tr}} \left[ \left( \sum _{\lambda :~{\bar{H}}(\lambda ) \in [H(\rho )\pm \delta ] } \varPi _{A^n}^\lambda \right) \rho _A^{\otimes n} \right] \geqslant {\text {tr}} \left[ \left( \sum _{\lambda \in \varLambda _{\delta '}} \varPi _{A^n}^\lambda \right) \rho _A^{\otimes n} \right] \end{aligned}$$

because all terms in the sum in the right hand side are included in the sum on the left hand side. We may now invoke [24, Eq. (6.23)] to see that

128 $$\begin{aligned} (128) \geqslant 1 - {\text {poly}}(n)\exp \left\{ - n\eta \right\} \ , \end{aligned}$$

where $$\eta =\delta '^2/2$$. $$\square $$

### Proof

(Proposition 2.2). The fact that there are only ${\text {poly}}(n)$ elements follows because there are only so many types. Property (ii) holds by definition. Property (iv) holds because $e^{-n(k\pm \delta )}$ is the minimum / maximum eigenvalue of $\Gamma _A^{\otimes n}$ in the subspace spanned by $R^{\approx _\delta h}_{A^n}$. Finally, we need to show Property (iii): This follows from a large deviation analysis. More precisely, let $Z_j$ for $j=1,\ldots ,n$ be random variables where $Z_j$ represents the measurement outcome of $H_A$ on the *j*-th system of the i.i.d. state $\rho _A^{\otimes n}$. By Hoeffding’s inequality, we have that

129 $$\begin{aligned} \Pr \left[ \left|{(1/n) \sum Z_j - {\text {tr}}[\rho _A H_A]}\right|> \delta \right]&\leqslant 2\exp \left( -\frac{2n\delta ^2}{\varDelta H_A^2}\right) \leqslant 2\exp \left( -\frac{n\delta ^2}{2\,\Vert {H_A}\Vert _\infty ^2}\right) \ , \end{aligned}$$

where $\varDelta H_A$ is the difference between the maximum and minimum eigenvalue of $H_A$, and $\varDelta H_A \leqslant 2\Vert {H_A}\Vert _\infty$. Thus, the event consisting of the outcomes *k* satisfying $|{k-{\text {tr}}[\rho _A H_A]}| \leqslant \delta$ happens with probability at least $1-2{e}^{-n\eta }$, proving (16). $\square$

### Proof

(Proposition 2.3) We use the post-selection technique (Theorem 2.1) to bound the diamond norm distance between ${\mathscr {T}}_{X^n\rightarrow X'^n}$ and ${\mathscr {E}}_{X\rightarrow X'}^{\otimes n}$. Let $|{\zeta }\rangle _{X^n{\bar{R}}^n R'}$ be the purification of the de Finetti state given by (21). Calculate

130 $$\begin{aligned}&{\text {Re}} \left\{ \langle {\zeta }|_{X^n{\bar{R}}^n R'} (V_{X\rightarrow EX'}^{\otimes n})^\dagger W_{X^n\rightarrow E^nX'^n} |{\zeta }\rangle _{X^n{\bar{R}}^n R'} \right\} \nonumber \\&\quad = \sum p_i\, {\text {Re}} \left\{ \langle {\phi _i}|_{X{\bar{R}}}^{\otimes n} \, (V_{X\rightarrow EX'}^{\otimes n})^\dagger \, W_{X^n\rightarrow E^nX'^n} |{\phi _i}\rangle _{X{\bar{R}}}^{\otimes n} \right\} \nonumber \\&\quad \geqslant 1 - {\text {poly}}(n)\exp (-n\eta ) \end{aligned}$$

which implies, recalling that $F(|{\psi }\rangle ,|{\phi }\rangle ) = |{\langle {\psi }\vert {\phi }\rangle }|\geqslant {\text {Re}}\{\langle {\psi }\vert {\phi }\rangle \}$ and that $(1-x)^2 \geqslant 1 - 2x$,

131 $$\begin{aligned} F^2 \left( V_{X\rightarrow EX'}^{\otimes n}\, |{\zeta }\rangle _{X^n{\bar{R}}^n R'} \, , \, W_{X^n\rightarrow E^nX'^n} |{\zeta }\rangle _{X^n{\bar{R}}^n R'} \right) \geqslant 1 - {\text {poly}}(n) \exp (-n\eta ) \end{aligned}$$

and hence

132 $$\begin{aligned} P \left( V_{X\rightarrow EX'}^{\otimes n}\, |{\zeta }\rangle _{X^n{\bar{R}}^n R'} \, , \, W_{X^n\rightarrow E^nX'^n} |{\zeta }\rangle _{X^n{\bar{R}}^n R'} \right) \leqslant {\text {poly}}(n) \exp (-n\eta /2)\ . \end{aligned}$$

Recalling the relations between the trace distance and the purified distance, and noting that these distance measures cannot increase under the partial trace, we obtain

133 $$\begin{aligned}&D\bigl ( {\mathscr {T}}(\zeta _{X^n{\bar{R}}^n R'}) , {\mathscr {E}}^{\otimes n}(\zeta _{X^n{\bar{R}}^n R'}) \bigr ) \leqslant P\bigl ( {\mathscr {T}}(\zeta _{X^n{\bar{R}}^n R'}) \;,\; {\mathscr {E}}^{\otimes n}(\zeta _{X^n{\bar{R}}^n R'}) \bigr ) \nonumber \\&\quad \leqslant P\bigl ( W_{X^n\rightarrow E^nX'^n} |{\zeta }\rangle _{X^n{\bar{R}}^n R'} \;,\; V_{X\rightarrow EX'}^{\otimes n}\, |{\zeta }\rangle _{X^n{\bar{R}}^n R'} \bigr ) \leqslant {\text {poly}}(n) \exp (-n\eta /2).\nonumber \\ \end{aligned}$$

The post-selection technique then asserts that

134 $$\begin{aligned} \frac{1}{2}\Vert { {\mathscr {T}} - {\mathscr {E}}^{\otimes n} }\Vert _\diamond \leqslant {\text {poly}}(n)\,\exp (-n\eta /2) \end{aligned}$$

as claimed. $$\square $$

### Proof

(Lemma 7.2). Let $V'_{X\rightarrow XE}$ be any Stinespring dilation isometry of ${\mathscr {E}}_{X\rightarrow X}$, such that ${\mathscr {E}}_{X\rightarrow X}(\cdot ) = {\text {tr}}_E \left[ V'_{X\rightarrow XE}\,(\cdot )\,V'^\dagger \right]$. For the input state $|{\varPhi }\rangle _{X:R_X}$, consider the output state $|{\varphi }\rangle _{XER_X}$ corresponding to first time-evolving by some time *t*, and then applying $V'$

135 $$\begin{aligned} |{\varphi }\rangle _{XER_X} = V'\,{e}^{-i H_X t}\,|{\varPhi }\rangle _{X:R_X} = {e}^{-i V' H_X V'^\dag t}\, V'\, |{\varPhi }\rangle _{X:R_X}\ . \end{aligned}$$

Now, let us define $|{\varphi '}\rangle _{XER_X} = {e}^{-i H_X t} \, V'\,|{\varPhi }\rangle _{X:R_X}$. By the covariance property of ${\mathscr {E}}_{X\rightarrow X}$ both $|{\varphi }\rangle$ and $|{\varphi '}\rangle$ have the same reduced state on $X R_X$. Hence, they are related by some unitary $W_E^{(t)}$ on the system *E* which in general depends on *t*

136 $$\begin{aligned} |{\varphi }\rangle _{XER_X} = W^{(t)}_E \, |{\varphi '}\rangle _{XER_X}\ . \end{aligned}$$

We have

137 $$\begin{aligned} {\text {tr}}_{X} \left[ V' {e}^{-iH_X t} \varPhi _{X:R_X} {e}^{i H_X t} V'^\dagger \right] = W^{(t)}_{E} {\text {tr}}_X\bigl [ V' \varPhi _{X:R_X} V'^\dagger \bigr ] W^{(t)\,\dagger }_{E} \end{aligned}$$

so $W^{(t)}_E$ must define a representation of time evolution, at least on the support of the operator ${\text {tr}}_X\bigl [ V' \varPhi _{X:R_X} V'^\dagger \bigr ]$. Hence, we may write $W^{(t)}_E = {e}^{-i H_E t}$ for some Hamiltonian $H_E$, and from (137), we have for all *t*

138 $$\begin{aligned} V'_{X\rightarrow XE} \, {e}^{-i H_{X} t} = {e}^{-i (H_X + H_E) t} \, V'_{X\rightarrow XE}\ . \end{aligned}$$

Expanding for infinitesimal *t* we obtain

139 $$\begin{aligned} V'_{X\rightarrow XE} \, H_X = \left( H_X + H_E \right) \, V'_{X\rightarrow XE}\ . \end{aligned}$$

Let $|{0}\rangle _E$ be an eigenvector of $H_E$ corresponding to the eigenvalue zero; if $H_E$ does not contain an eigenvector with eigenvalue equal to zero, we may trivially add a dimension to the system *E* to accommodate this vector. Then, the operator $V'_{X\rightarrow XE} \langle {0}|_E$ maps each state of a subset of energy levels of *XE* to a corresponding energy level of same energy on *XE*; it may thus be completed to a fully energy-preserving unitary $V_{XE\rightarrow XE}$. More precisely, let $|{j}\rangle _X$ be a complete set of eigenvectors of $H_X$ with energies $h_j$. Then $|{\psi _j'}\rangle = V'_{X\rightarrow XE}|{j}\rangle _X$ is an eigenvector of $H_X+H_E$ of energy $h_j$ thanks to (140). We have two orthonormal sets $\bigl \{ |{0}\rangle _E\otimes |{j}\rangle _X \bigr \}$ and $\bigl \{ |{\psi _j'}\rangle _X \bigr \}$ in which the *j*-th vector of each set has the same energy; we can thus complete these sets into two bases $\left\{ |{\chi _i}\rangle _{XE} \right\}$, $\left\{ |{\chi '_i}\rangle _{XE} \right\}$ of eigenvectors of $H_X+H_E$, where the *i*-th element of either basis has exactly the same energy. This defines a unitary $V_{XE\rightarrow XE} = \sum _i |{\chi '_i}\rangle _{XE}\langle {\chi _i}|_{XE}$ that is an extension of $V'_{X\rightarrow XE} \langle {0}|_E$, and that satisfies all the conditions of the claim. $\square$

## B. Technical Lemmas

### Lemma B.1

(Pinching-like operator inequality). Let $\{E^i\}_{i=1}^M$ be a collection of *M* operators and $T\geqslant 0$. Then, we have

140 $$\begin{aligned} \left( \sum E^i\right) \, T\, \left( \sum E^{j\,\dagger }\right) \leqslant M \sum E^i\,T\,E^{i\,\dagger }\ . \end{aligned}$$

### Proof

Call our system *S* and consider an additional register *C* of dimension $|{C}|= M$, and let $|{\chi }\rangle _C = M^{-1/2}\sum _{k=1}^M |{k}\rangle _C$. Then, we have

141 $$\begin{aligned} \left( \sum E_S^i\right) \, T_S\, \left( \sum E_S^{j\,\dagger }\right)&= {\text {tr}}_C \left[ \left( \sum E_S^i\otimes |{i}\rangle _C\right) \, T_S\, \left( \sum E_S^{j\,\dagger }\otimes \langle {j}|_C\right) \, \left( \mathbb {1}_S\otimes (M\,|{\chi }\rangle \langle {\chi }|_C) \right) \right] \nonumber \\&\leqslant M {\text {tr}}_C \left[ \left( \sum E_S^i\otimes |{i}\rangle _C\right) \, T_S\, \left( \sum E_S^{j\,\dagger }\otimes \langle {j}|_C\right) \, \left( \mathbb {1}_S\otimes \mathbb {1}_C\right) \right] \nonumber \\&= M \sum E_S^i \, T_S\, E_S^{i\,\dagger }\ , \end{aligned}$$

using $$|{\chi }\rangle \langle {\chi }|_C\leqslant \mathbb {1}_C$$. $$\square $$

### Lemma B.2

(Gentle measurement). Let $\rho$ be a sub-normalized quantum state and $0\leqslant Q\leqslant \mathbb {1}$. For ${\text {tr}}[Q\rho ]\geqslant 1 - \delta$ we then have

142 $$\begin{aligned} P(\rho , Q^{1/2}\rho Q^{1/2}) \leqslant \sqrt{2\delta }\ . \end{aligned}$$

This is a cruder statement than that of, e.g., [69, Lemma 7], allowing for a more straightforward proof.

### Proof

We have

143 $$\begin{aligned} {\bar{F}}(\rho , Q^{1/2}\rho Q^{1/2}) \geqslant F(\rho , Q^{1/2}\rho Q^{1/2})&= {\text {tr}}\left[ \sqrt{\rho ^{1/2}(Q^{1/2}\rho Q^{1/2})\rho ^{1/2}}\right] \nonumber \\&= {\text {tr}}\left[ Q^{1/2}\rho \right] \geqslant {\text {tr}}[Q\rho ] \geqslant 1 - \delta \ . \end{aligned}$$

Then, we get $P(\rho , Q^{1/2}\rho Q^{1/2}) \leqslant \sqrt{ 1 - (1 - \delta )^2 } \leqslant \sqrt{2\delta }$. $\square$

### Proposition B.3

(Controlled-unitary using a POVM). Let $\{ Q^j \}$ be a set of positive semi-definite operators on a system *X* satisfying $\sum Q^j \leqslant \mathbb {1}$, $\{ U^j \}$ be a collection of unitaries on a system *Y*, and

144 $$\begin{aligned} W_{XY} = \sum _j Q_X^j \otimes U_Y^j\ . \end{aligned}$$

Then, we have $W^\dagger W \leqslant \mathbb {1}$.

### Proof

Using an additional register *K*, define

145 $$\begin{aligned} V_{X\rightarrow XK} = \sum \sqrt{Q^j} \otimes |{j}\rangle _K\ . \end{aligned}$$

Then, we have $V^\dagger V = \sum Q^j \leqslant \mathbb {1}$. Clearly, $VV^\dagger \leqslant \mathbb {1}_{XK}$ because $VV^\dagger$ and $V^\dagger V$ have the same non-zero eigenvalues. Now, let

146 $$\begin{aligned} W = V^\dagger \left( \sum \mathbb {1}_X\otimes U^j_Y \otimes |{j}\rangle \langle {j}|_K\right) \, V\ . \end{aligned}$$

Because the middle term in parentheses is unitary, we manifestly have $W^\dagger W \leqslant \mathbb {1}$. $\square$

## C. Dilation of Energy-Conserving Operators to Unitaries

This appendix collects a few technical lemmas on constructing an energy-conserving unitary that extends a given operator of norm less than one.

### Proposition C.1

Let $W_X$ be an operator on a system *X*, such that $W^\dagger W \leqslant \mathbb {1}$. Then, there exists a unitary operator $U_{XQ}$ acting on *X* and a qubit *Q* such that for any $|{\psi }\rangle _X$,

147 $$\begin{aligned} \langle {0}|_Q\,U_{XQ}\,(|{\psi }\rangle _X\otimes |{0}\rangle _Q) = W_{X} |{\psi }\rangle _X\ . \end{aligned}$$

That is, any operator *W* with $\Vert {W}\Vert _\infty \leqslant 1$ can be dilated to a unitary, with a post-selection on the output.

### Proof

Setting $V_{X\rightarrow XQ} = W\otimes |{0}\rangle _Q + \sqrt{\mathbb {1}- W^\dagger {}W} \otimes |{1}\rangle _Q$, we see that $V^\dagger V = W^\dagger W + \mathbb {1}- W^\dagger W = \mathbb {1}_X$, and hence $V_{X\rightarrow XQ}$ is an isometry. We can complete this isometry to a unitary $U_{XQ}$ that acts as *V* on the support of $\mathbb {1}_X\otimes |{0}\rangle \langle {0}|_Q$ and that maps the the support of $\mathbb {1}_X\otimes |{1}\rangle \langle {1}|_Q$ onto the complementary space to the image of *V*. It then follows that for any $|{\psi }\rangle _X$, we have $U_{XQ}\,(|{\psi }\rangle _X\otimes |{0}\rangle _Q) = V_{X\rightarrow XQ}\, |{\psi }\rangle _X = (W_X|{\psi }\rangle _X)\otimes |{0}\rangle _Q + (\ldots )\otimes |{1}\rangle _Q$, and the claim follows. $\square$

### Proposition C.2

Let *X* be a quantum system with Hamiltonian $H_X$ and $W_X$ be an operator with $W^\dagger W \leqslant \mathbb {1}$ as well as $[W_X, H_X] = 0$. Then, there exists a unitary operator $U_{XQ}$ acting on *X* and a qubit *Q* with $H_Q=0$, that satisfies $[U_{XQ}, H_X] = 0$ such that

148 $$\begin{aligned} \langle {0}|_Q\,U_{XQ}\,|{0}\rangle _Q = W_{X}\ . \end{aligned}$$

That is, any energy-preserving operator *W* with $\Vert {W}\Vert _\infty \leqslant 1$ can be dilated to an energy-preserving unitary on an ancilla with a post-selection on the output.

### Proof

First we calculate $[W^\dagger W, H_X] = W^\dagger [W, H_X] + [W^\dagger , H_X]\, W = 0 - [W, H_X]^\dagger W = 0$. This implies that $[\sqrt{\mathbb {1}- W^\dagger W}, H_X] = 0$, as $W^\dagger W$ and $\sqrt{\mathbb {1}- W^\dagger W}$ have the same eigenspaces. We define

149 $$\begin{aligned} V_{X\rightarrow XQ} = W\otimes |{0}\rangle _Q + \sqrt{\mathbb {1}- W^\dagger {}W} \otimes |{1}\rangle _Q\ . \end{aligned}$$

The operator $V_{X\rightarrow XQ}$ is an isometry, because $V^\dagger V = W^\dagger W + \mathbb {1}- W^\dagger W = \mathbb {1}_X$. Furthermore, we have

150 $$\begin{aligned} V_{X\rightarrow XQ} \, H_X&= (W_X H_X)\otimes |{0}\rangle + (\sqrt{\mathbb {1}- W^\dagger W}\, H_X)\otimes |{1}\rangle \end{aligned}$$

151 $$\begin{aligned}&= (H_X W_X)\otimes |{0}\rangle + (H_X \sqrt{\mathbb {1}- W^\dagger W}) \otimes |{1}\rangle = H_X \, V_{X\rightarrow XQ} \end{aligned}$$

and thus we find $[V_{X\rightarrow XQ}, H_X] = 0$. Let $\bigl \{ |{j}\rangle _X \bigr \}$ be an eigenbasis of $H_X$, and let $|{\psi '_j}\rangle _{XQ} = V_{X\rightarrow XQ} |{j}\rangle _X$, noting that both $|{j}\rangle _X$ and $|{\psi '_j}\rangle _{XQ}$ have the same energy. The two collections of vectors $\bigl \{ |{j}\rangle _X\otimes |{0}\rangle _Q \bigr \}$ and $\bigl \{ |{\psi '_j}\rangle _{XQ} \bigr \}$ can thus be completed into two bases $\bigl \{ |{\chi _i}\rangle _{XQ} \bigr \}$ and $\bigl \{ |{\chi '_i}\rangle _{XQ} \bigr \}$ of eigenvectors of $H_X + H_Q$ where the *i*-th element of both bases have the same energy. Define finally $U_{XQ} = \sum _i |{\chi '_i}\rangle \langle {\chi _i}|_{XQ}$, noting that by construction $U_{XQ}|{0}\rangle _Q = V_{X\rightarrow XQ}$ and $[U_{XQ}, H_X] = 0$. $\square$

## D. Robust Counterexample Against Extensions of Construction #1

In this appendix we show that the counterexample of Sect. 5.2 is robust to small errors on the process. The process is ${\mathscr {E}}_{X\rightarrow X'}(\cdot ) = {\text {tr}}[\cdot ]|{+}\rangle \langle {+}|$, where $|{+}\rangle = [|{0}\rangle +|{1}\rangle ]/\sqrt{2}$ with $|{0}\rangle ,|{1}\rangle$ energy eigenstates of respective energies $E_0=0$, $E_1>0$; we write $H_X = \sum _{j=0,1} E_j |{j}\rangle \langle {j}|$ and $\Gamma _X = {e}^{-\beta H_X}$. The initial state on *X* and a reference system $R_X\simeq X$ is the maximally entangled state $|{\sigma }\rangle _{XR_X} = [|{00}\rangle + |{11}\rangle ]/\sqrt{2} = |{\varPhi }\rangle _{X:R_X}/\sqrt{2}$.

We seek a map ${\mathscr {T}}_{X\rightarrow X'}$ such that

152 $$\begin{aligned} P({\mathscr {T}}_{X\rightarrow X'}(\sigma _{XR_X}), {\mathscr {E}}_{X\rightarrow X'}(\sigma _{XR_X})) \leqslant \epsilon \qquad \text {and}\qquad {\mathscr {T}}_{X\rightarrow X}(\Gamma _X)\leqslant \alpha \Gamma _{X'}\ , \end{aligned}$$

for a $\alpha$ that is independent of $E_0,E_1$. Here we have $X\simeq X'$ and $\Gamma _X=\Gamma _{X'}$.

Let $\rho _{X'R_X} = {\mathscr {E}}_{X\rightarrow X'}(\sigma _{XR_X})$. From (153) we find $\frac{1}{2} \Vert {{\mathscr {T}}_{X\rightarrow X'}(\sigma _{XR_X}) - \rho _{X'R_X}}\Vert _1 \leqslant \epsilon$, which in turn implies that $(1/4)\bigl \Vert {\mathscr {T}}_{X\rightarrow X'}(\varPhi _{X:R_X}) - |{+}\rangle \langle {+}|_{X'}\otimes \mathbb {1}_{R_X}\bigr \Vert _1 \leqslant \epsilon$, and hence that ${\mathscr {T}}_{X\rightarrow X'}(\cdot ) = {\text {tr}}[\cdot ]\,|{+}\rangle \langle {+}|_{X'} + \varDelta (\cdot )$ for some Hermiticity preserving map $\varDelta (\cdot )$ satisfying $\frac{1}{2}\Vert {\varDelta (\varPhi _{XR_X})}\Vert _1\leqslant 2\epsilon$.

Let $\varDelta _\pm \geqslant 0$ be the positive and negative parts of $\varDelta (\Gamma ) = \varDelta _+ - \varDelta _-$, noting that ${\text {tr}}(\varDelta _-) \leqslant {\text {tr}}(\varDelta _-) + {\text {tr}}(\varDelta _+) = \Vert {\varDelta (\Gamma )}\Vert _1 = \Vert {{\text {tr}}_{R_X}\bigl ( \Gamma _{R_X}^{1/2}\,\varDelta (\varPhi _{X:R_X})\,\Gamma _{R_X}^{1/2} \bigr ) }\Vert _1$, defining $\Gamma _{R_X}$ as the transpose of $\Gamma _X$ onto the system $R_X$, and continuing the computation we obtain ${\text {tr}}(\varDelta _-) \leqslant \Vert {\Gamma _{R_X}^{1/2}\,\varDelta (\varPhi _{X:R_X})\,\Gamma _{R_X}^{1/2} }\Vert _1 \leqslant \Vert {\Gamma _{R_X}}\Vert _\infty \Vert { \varDelta (\varPhi _{X:R_X}) }\Vert _1 \leqslant 4\epsilon$, using the fact that $\Vert {\Gamma _X}\Vert _\infty = \max _j \{ {e}^{-\beta E_j} \} = 1$.

To complete this argument we define the hypothesis testing relative entropy [70–74] in its form as presented in [75]. For any sub-normalized quantum state $\rho$ and for any positive semi-definite operator $\sigma$ whose support contains the support of $\rho$, we define it via the following equivalent optimizations, which are semi-definite programs [76] in terms of the primal variable $Q\geqslant 0$ and the dual variables $\mu , X\geqslant 0$:

153 $$\begin{aligned} \begin{array}{lllllll} {e}^{ -{D}_{\mathrm {H}}^{\eta }(\rho \,\Vert \,\sigma ) } &{}=&{} \text {minimize:}&{} \eta ^{-1}\,{\text {tr}}[Q\sigma ]&{} =&{}\text {maximize:} &{} \mu - \eta ^{-1}{\text {tr}}[X]\\ &{}&{}\text {subject\ to:}&{}Q \leqslant \mathbb {1}&{}=&{}\text {subject to:}&{} \mu \rho \leqslant \sigma + X.\\ &{}&{}&{} {\text {tr}}[Q\rho ] \geqslant \eta &{}&{}&{}\\ \end{array} \end{aligned}$$

The condition ${\mathscr {T}}_{X\rightarrow X'}(\Gamma ) \leqslant \alpha \Gamma$ implies that $\alpha \Gamma \geqslant {\text {tr}}[\Gamma ]|{+}\rangle \langle {+}|+ \varDelta (\Gamma ) \geqslant |{+}\rangle \langle {+}|- \varDelta _-$. Hence, we have that $\alpha ^{-1}\,|{+}\rangle \langle {+}|\leqslant \Gamma + \varDelta _-/\alpha$. Hence, for any $0<\eta \leqslant 1$ to be fixed later, $\mu =\alpha ^{-1}$ is feasible for the dual problem (154) defining the hypothesis testing entropy ${D}_{\mathrm {H}}^{\eta }(|{+}\rangle \langle {+}|\,\Vert \,\Gamma )$, and ${e}^{-{D}_{\mathrm {H}}^{\eta }(|{+}\rangle \langle {+}|\,\Vert \,\Gamma )} \geqslant \alpha ^{-1} - {\text {tr}}[\varDelta _-/\alpha ]/\eta \geqslant \alpha ^{-1} \bigl ( 1 - 4\epsilon /\eta \bigr )$. Thus, we have $\ln (\alpha ) \geqslant {D}_{\mathrm {H}}^{\eta }(|{+}\rangle \langle {+}|\,\Vert \,\Gamma ) + \ln (1 - 4\epsilon /\eta )$. Choosing $\eta =8\epsilon$ yields $\ln (1 - 4\epsilon /\eta ) = -\ln (2)$.

On the other hand, by definition we have ${e}^{-{D}_{\mathrm {H}}^{\eta }(|{+}\rangle \langle {+}|\,\Vert \,\Gamma )} \leqslant {\text {tr}}[Q\Gamma ]/\eta$ for any $0\leqslant Q\leqslant \mathbb {1}$ satisfying ${\text {tr}}[Q|{+}\rangle \langle {+}|]\geqslant \eta$; with $Q=2\eta |{1}\rangle \langle {1}|$ we obtain ${e}^{-{D}_{\mathrm {H}}^{\eta }(|{+}\rangle \langle {+}|\,\Vert \,\Gamma )} \leqslant 2{e}^{-\beta E_1}$ and thus ${D}_{\mathrm {H}}^{\eta }(|{+}\rangle \langle {+}|\,\Vert \,\Gamma ) \geqslant \beta E_1 -\ln (2)$.

Then, $\ln (\alpha ) \geqslant -\ln (2) + \beta E_1 -\ln (2) = -2\ln (2) + \beta E_1$. Now let $\alpha$ be the optimal candidate in the coherent relative entropy ${\hat{D}}_{X\rightarrow X'}^{\epsilon }(\rho _{X'R_{X}}\,\Vert \,\Gamma ,\,\Gamma ) = -\ln (\alpha )$. We finally see that the transformation $\mathbb {1}/2\rightarrow |{+}\rangle$ may require arbitrarily much energy if $E_1\rightarrow \infty$, even for a small $\epsilon >0$, since

154 $$\begin{aligned} \text {energy cost} = - \beta ^{-1}{\hat{D}}_{X\rightarrow X'}^{\epsilon }(\rho _{X'R_{X}}\,\Vert \,\Gamma ,\,\Gamma ) = \beta ^{-1}\ln (\alpha ) \geqslant E_1 - 2 \beta ^{-1} \ln (2)\ . \end{aligned}$$

## E. Universal Conditional Typical Projector for Trivial Hamiltonians

In the case of trivial Hamiltonians, Definition 6.1 can be simplified. We call the corresponding object a universal conditional typical projector

### Definition E.1

Consider two systems with Hilbert spaces ${\mathscr {H}}_A,{\mathscr {H}}_B$ and let $s\in {\mathbb {R}}$. We define a *universal conditional typical projector* $P^{s,\delta }_{A^nB^n}$ with parameter $\delta >0$ as a projector acting on $({\mathscr {H}}_A\otimes {\mathscr {H}}_B)^{\otimes n}$ such that:

(i) There exists $\eta >0$ independent of *n* such that for any quantum state $\rho _{AB}$ with ${H}(A\,|\,B)_{\rho }\leqslant s$, we have
  155 $$\begin{aligned} {\text {tr}}\bigl [P^{s,\delta }_{A^nB^n}\,\rho _{AB}^{\otimes n}\bigr ] \geqslant 1 - {\text {poly}}(n)\exp (-n\eta )\ ; \end{aligned}$$
(ii) $$\displaystyle {\text {tr}}_{A^n}\bigl [ P^{s,\delta }_{A^nB^n} \bigr ] \leqslant {\text {poly}}(n)\,{e}^{n (s + 2\delta )}\,\mathbb {1}_{B^n}$$.

Observe that we choose to define the object in Definition E.1 as a projector whereas we only require the object in Definition 6.1 to be an operator of norm at most 1. The reason is that while we can prove that a projector satisfying the conditions of Definition E.1 exists, we are currently not able to guarantee the existence of a projector satisfying the criteria of Definition 6.1.

### Proposition E.2

Consider two systems *A*, *B* and let $s\in {\mathbb {R}}$. For any $\delta >0$ and $n\in {\mathbb {N}}$ there exists a universal conditional typical projector $P_{A^nB^n}^{s,\delta }$ that is permutation-invariant.

The proof of Proposition E.2 is developed in the rest of this appendix. To understand why the projector of Definition E.1 is conditional—as well as for a simple illustration of its use—consider the smooth Rényi-zero conditional max-entropy, also known as the smooth alternative max-entropy [11]. It is defined for a bipartite state $\rho _{AB}$ as

156 $$\begin{aligned} {\hat{H}}_{\mathrm {max}}^{\epsilon }(A\,|\,B)_{\rho } = \min _{{\hat{\rho \approx _\epsilon \rho }}} \, \ln \,\bigl \Vert { {\text {tr}}_A\bigl [ \varPi ^{{\hat{\rho }}_{AB}}_{AB} \bigr ] }\bigr \Vert _\infty \ , \end{aligned}$$

where $\varPi ^{{\hat{\rho }}_{AB}}_{AB}$ is the projector onto the support of ${\hat{\rho }}_{AB}$, and where the optimization ranges over sub-normalized states ${\hat{\rho }}_{AB}$ which are $\epsilon$-close to $\rho _{AB}$ in purified distance. We may understand the i.i.d. behaviour of this quantity as follows. For $\delta >0$ and $n\in {\mathbb {N}}$ let $P^{s,\delta }_{A^nB^n}$ be a universal conditional typical projector with $s={H}(A\,|\,B)_{\rho }$. We define ${\hat{\rho }}_{A^nB^n} = P^{s,\delta }\,\rho _{AB}^{\otimes n}\,P^{s,\delta }$. Then, we have ${\hat{\rho }}_{A^nB^n} \approx _\epsilon \rho _{AB}^{\otimes n}$ for $n\in {\mathbb {N}}$ large enough, thanks to Property (i) and the gentle measurement lemma (Lemma B.2). On the other hand, using Property (ii) we have

157 $$\begin{aligned} \frac{1}{n} {\hat{H}}_{\mathrm {max}}^{\epsilon }(A^n\,|\,B^n)_{\rho ^{\otimes n}} \leqslant \frac{1}{n} \ln \,\bigl \Vert { {\text {tr}}_{A^n} \bigl [ P^{s,\delta } \bigr ] }\bigr \Vert _\infty \leqslant {H}(A\,|\,B)_{\rho } + 2\delta + \frac{1}{n}\ln ({\text {poly}}(n)) \end{aligned}$$

such that taking the limits $n\rightarrow \infty$ and $\delta \rightarrow 0$, we get that the smooth Rényi-zero conditional entropy is asymptotically upper bounded by the von Neumann conditional entropy in the i.i.d. regime.

We proceed to construct a universal conditional typical projector based on ideas from Schur–Weyl duality. The construction presented here is similar to, and inspired by, techniques put forward in earlier work [22, 24–26, 47, 48].

### Proof

(Proposition E.2) Let

158 $$\begin{aligned} P^{s,\delta }_{A^nB^n} = \sum _{\begin{array}{c} \lambda ,\lambda '\;:\\ {\bar{H}}(\lambda ) - {\bar{H}}(\lambda ') \leqslant s + 2\delta \end{array}} (\mathbb {1}_{A^n}\otimes \varPi ^{\lambda '}_{B^n})\,\varPi ^{\lambda }_{A^nB^n}\ , \end{aligned}$$

where the respective projectors $\varPi ^{\lambda '}_{B^n}$, $\varPi ^{\lambda }_{A^nB^n}$ refer to Schur–Weyl decompositions of ${\mathscr {H}}_{B}^{\otimes n}$ and of $({\mathscr {H}}_{A}\otimes {\mathscr {H}}_B)^{\otimes n}$, respectively, $\lambda \in {\text {Young}}(d_Ad_B,n)$ and $\lambda '\in {\text {Young}}(d_{B},n)$. Observe that $P^{s,\delta }_{A^nB^n}$ is a projector: Each term in the sum is a projector as a product of two commuting projectors (Lemma 2.1), and each term of the sum acts on a different subspace of $({\mathscr {H}}_A\otimes {\mathscr {H}}_B)^{\otimes n}$. The projector $P^{s,\delta }_{A^nB^n}$ corresponds to the measurement of the two commuting POVMs $\bigl \{ \varPi ^{\lambda }_{A^nB^n} \bigr \}$ and $\bigl \{ \varPi ^{\lambda '}_{B^n} \bigr \}$, and testing whether or not the event ${\bar{H}}(\lambda ) - {\bar{H}}(\lambda ') \leqslant s + 2\delta$ is satisfied. Also by construction $P^{s,\delta }_{A^nB^n}$ is permutation-invariant.

For any $\rho _{AB}$ with ${H}(A\,|\,B)_{\rho } \leqslant s$, the probability that the measurement of $P^{s,\delta }_{A^nB^n}$ fails on $\rho _{AB}^{\otimes n}$ can be upper bounded as follows. The passing event ${\bar{H}}(\lambda ) - {\bar{H}}(\lambda ') \leqslant s + 2\delta$ is implied in particular by the two events (a) ${\bar{H}}(\lambda ) \leqslant H(AB)_\rho + \delta$ and (b) ${\bar{H}}(\lambda ') \geqslant H(B)_\rho - \delta$ happening simultaneously, recalling that $H(AB)_\rho - H(B)_\rho = {H}(A\,|\,B)_{\rho } \leqslant s$. The probability of event (a) failing is

159 $$\begin{aligned} \Pr \left[ {\bar{H}}(\lambda ) > H(AB)_\rho + \delta \right] \leqslant {\text {poly}}(n) \exp \left( -n\eta \right) \end{aligned}$$

as given by Proposition 2.1, and similarly for event (b)

160 $$\begin{aligned} \Pr \left[ {\bar{H}}(\lambda ') < H(B)_\rho - \delta \right] \leqslant {\text {poly}}(n) \exp \left( -n\eta \right) \ . \end{aligned}$$

We can use the same $\eta$ in both cases by picking the lesser of the two values given by Proposition 2.1, if necessary. Note furthermore that $\eta >0$ does not depend on $\rho$. Hence with this $\eta$, for any $\rho _{AB}$ we have

161 $$\begin{aligned} {\text {tr}}\bigl [P^{s,\delta }_{A^nB^n}\, \rho _{AB}^{\otimes n}\bigr ] \geqslant 1 - {\text {poly}}(n)\exp (-n\eta ) \end{aligned}$$

as required.

For the second property, we use Lemma 2.2 to write

162 $$\begin{aligned} {\text {tr}}_{A^n}\bigl [ P^{s,\delta }_{A^nB^n} \bigr ]&= \sum _{\begin{array}{c} \lambda ,\lambda '\;:\\ {\bar{H}}(\lambda ) - {\bar{H}}(\lambda ') \leqslant s + 2\delta \end{array}} \varPi ^{\lambda '}_{B^n}\, {\text {tr}}_{A^n} \left[ \varPi ^{\lambda }_{A^nB^n}\right] \, \varPi ^{\lambda '}_{B^n} \nonumber \\&\leqslant \sum _{\begin{array}{c} \lambda ,\lambda '\;:\\ {\bar{H}}(\lambda ) - {\bar{H}}(\lambda ') \leqslant s + 2\delta \end{array}} {\text {poly}}(n)\,{e}^{n({\bar{H}}(\lambda ) - {\bar{H}}(\lambda '))} \mathbb {1}_{B^n} \nonumber \\&\leqslant {\text {poly}}(n)\,{e}^{n(s+2\delta )}\, \mathbb {1}_{B^n} \end{aligned}$$

recalling that there are only ${\text {poly}}(n)$ many possible Young diagrams and hence at most so many terms in the sum. $\square$

## F. Universal Conditional Erasure for *n* Copies and Trivial Hamiltonians

### Corollary F.1

(Thermodynamic protocol for universal conditional erasure for n copies). Let *S*, *M* be systems, let $\sigma _{S}$ be the maximally mixed state on *S*. Let $s < \ln (d_S)$, where $d_S$ is the dimension of *S*, and let $\delta >0$ small enough. Let $n\in {\mathbb {N}}$ be large enough. Let *J* be a large enough information battery and let any $m \leqslant n(\ln (d_S) - s - 3\delta )$ such that $e^m$ is integer.

Then, there exists $\eta '>0$ and a thermal operation ${\mathscr {R}}_{S^nM^nJ\rightarrow S^nM^nJ}$ acting on the systems $S^nM^nJ$, such that the effective work process ${\mathscr {T}}_{S^nM^n\rightarrow S^nM^n}$ of ${\mathscr {R}}_{S^nM^nJ\rightarrow S^nM^nJ}$ with respect to the battery states $(\tau _J^m,|{0}\rangle _J)$ is a universal conditional $({\text {poly}}(n)\,{e}^{-n\eta '})$-erasure process resetting $S^n$ to the state $\sigma _{S}^{\otimes n}$ with respect to the set of states ${\mathscr {S}}_{S^nM^n}'$, where ${\mathscr {S}}_{S^nM^n}'$ is the convex hull of ${\mathscr {S}}_{S^nM^n} = \bigl \{ \rho _{SM}^{\otimes n} : {H}(S\,|\,M)_{\rho } \leqslant s \bigr \}$.

The case where $s = \ln (d_S)$ is uninteresting as we cannot hope to extract any work. In such cases one can simply set $m=0$ and take ${\mathscr {R}}_{S^nM^nJ}$ to be the thermal operation that completely thermalizes $S^n$.

### Proof

This is in fact a relatively straightforward application of Proposition 7.1 over *n* copies of *SM*. Let $P_{S^nM^n}^{s,\delta }$ be given by Proposition E.2. We seek $\kappa ,\kappa '$ that satisfy (87). We can choose $\kappa = {\text {poly}}(n)\exp \left\{ -n\eta (\delta )\right\}$ thanks to Definition E.1. Furthermore for any $\rho _{SM}^{\otimes n}\in {\mathscr {S}}_{S^nM^n}$ we have

163 $$\begin{aligned} {\text {tr}} \left[ P_{S^nM^n} \left( \frac{\mathbb {1}_S}{d_S}\otimes \rho _M\right) ^{\otimes n}\right] \leqslant {\text {poly}}(n)\,{e}^{n(s+2\delta )}\,d_S^{-n}\,{\text {tr}}[\rho _M^{\otimes n}]&= {\text {poly}}(n)\,{e}^{-n(\ln (d_S) - s - 2\delta )}\nonumber \\&\leqslant \frac{{\text {poly}}(n)\,{e}^{-n\delta }}{{e^m}} \end{aligned}$$

and thus we may take $\kappa ' = {\text {poly}}(n)\,{e}^{-n\delta }$. Finally, $\eta '$ is given as $\eta '=\min \{\delta ,\eta (\delta )\}$. $\square$
